# Effects of water movement and temperature on *Rhizophydium* infection of *Planktothrix* in a shallow hypereutrophic lake

**DOI:** 10.3389/fmicb.2023.1197394

**Published:** 2023-06-19

**Authors:** Ryan S. Wagner, Katelyn M. McKindles, George S. Bullerjahn

**Affiliations:** ^1^Department of Biology, Bowling Green State University, Bowling Green, OH, United States; ^2^Great Lakes Center for Fresh Waters and Human Health, Bowling Green State University, Bowling Green, OH, United States; ^3^Ecology and Evolutionary Biology, College of Literature, Science, and the Arts, University of Michigan, Ypsilanti, MI, United States

**Keywords:** Chytridiomycota, *Planktothrix agardhii*, harmful algal blooms, food web, microcystin, mesocosms

## Abstract

Grand Lake St. Marys (GLSM) is a popular recreational lake located in western Ohio, United States, generating nearly $150 million in annual revenue. However, recurring algal blooms dominated by *Planktothrix agardhii*, which can produce harmful microcystin toxins, have raised concerns about water safety and negatively impacted the local economy. *Planktothrix agardhii* is host to a number of parasites and pathogens, including an obligate fungal parasite in the Chytridiomycota (chytrids). In this study, we investigated the potential of these chytrid (*Rhizophydium* sp.) to infect *P. agardhii* blooms in the environment by modifying certain environmental conditions thought to limit infection prevalence in the wild. With a focus on temperature and water mixing, mesocosms were designed to either increase or decrease water flow compared to the control (water outside the mesocosm). In the control and water circulation mesocosms, infections were found infrequently and were found on less than 0.75% of the *Planktothrix* population. On the other hand, by decreasing the water flow to stagnation, chytrid infections were more frequent (found in nearly 3x as many samples) and more prevalent, reaching a maximum infection rate of 4.12%. In addition, qPCR coupled with 16S–18S sequencing was utilized to confirm the genetic presence of both host and parasite, as well as to better understand the effect of water circulation on the community composition. Statistical analysis of the data confirmed that chytrid infection was dependent on water temperature, with infections predominantly occurring between 19°C and 23°C. Additionally, water turbulence can significantly reduce the infectivity of chytrids, as infections were mostly found in stagnant mesocosms. Further, decreasing the water circulation promoted the growth of the cyanobacterial population, while increasing water agitation promoted the growth of green algae (Chlorophyta). This study starts to explore the environmental factors that affect chytrid pathogenesis which can provide valuable insights into controlling measures to reduce the prevalence of harmful algal blooms and improve water quality in GLSM and similarly affected waterbodies.

## 1. Introduction

Cyanobacterial blooms are increasing with global climate and land use changes. As a result of large-scale land development and conversion to agriculture, nutrient loading into our waters has increased the size and duration of cyanobacterial harmful algal blooms (cHABS; Paerl and Barnard, 2020; Paerl et al., 2020). Furthermore, climate change has altered weather patterns, creating longer growing periods that, when coupled with increases in temperatures, favor cHABs (Paerl and Huisman, 2008). cHABs are of concern due to their ability to produce toxic compounds, known as cyanotoxins. These toxic metabolites have led to disruptions in drinking water and recreational uses (Bullerjahn et al., 2016). Understanding the mechanisms by which blooms form, are sustained, and decline are of great importance if bloom events are to be mitigated in the future.

*Planktothrix* is a cHAB species that is more competitive at lower light intensities and over a broader range of growth temperatures than other dominant cHAB species (Foy et al., 1976; Post et al., 1985). *Planktothrix*, with the help of gas vesicles, can adjust their buoyancy in the water column, allowing them to self-shade (Oberhaus et al., 2007) and are a filamentous cyanobacterium that is nutritionally inadequate (Schwarzenberger et al., 2020) to many zooplankton, meaning grazing pressures may be low. It is also adapt at nutrient acquisition by excreting alkaline phosphatases, allowing for the use of dissolved organic phosphorus when phosphate is depleted (Feuillade et al., 1990; Schwarzenberger et al., 2020), and is an excellent scavenger of nitrogen (Hampel et al., 2019). Thus, *Planktothrix* is ideally adapted to thriving under changes in light, temperature, and nutrient availability.

Whereas mechanisms of bloom decline involve both physical and biological drivers, fungal parasitism has received less attention in controlling cyanobacterial blooms compared to physicochemical factors and zooplankton (Sommer et al., 1986; Kagami et al., 2007). Chytridiomycota, often referred to as chytrids, is a phylum of fungi that can be aquatic parasites of phytoplankton. Chytrid infections during epidemics in freshwater environments may exceed 90% of host species (Kagami et al., 2006; Gsell et al., 2013b), particularly for hosts within the *Bacillariophyceae*. Parasitic chytrid infections within the *Cyanophyta* are less frequently linked to epidemics, but can still cause significant mortality largely because every infection leads to the death of the host (Rasconi et al., 2012; Gerphagnon et al., 2017; McKindles et al., 2021a,b). Parasitic chytrids are of great importance because they are involved in most trophic links within aquatic food webs and can contribute significantly to the transfer of carbon and energy between trophic levels through trophic upgrading of nutritionally important molecules (Amundsen et al., 2009; Miki et al., 2011; Gerphagnon et al., 2019). This transfer of carbon and energy has been observed with the bloom-forming cyanobacterium *Planktothrix* and its chytrid parasite (Frenken et al., 2018), and in other host-chytrid systems (Agha et al., 2016). Moreover, chytrids have the potential to regulate host populations, maintain host genetic diversity, and affect community structure (Sønstebø and Rohrlack, 2011; Kagami et al., 2014; Kyle et al., 2015). This host–parasite relationship has likely driven an evolutionary genetic response of the host population to chytrid infection, leading to strain specific susceptibility and the resistance to different chytrid isolates (McKindles et al., 2021a, 2023).

Temperature has a large effect on the rate of chytrid infection, both requiring an optimal temperature in which the host is viable and susceptible to infection, and an optimal temperature for the chytrid parasite to infect. To test this relationship, multiple isolates of *Planktothrix agardhii* and its parasite were obtained from the local water body Sandusky Bay, Lake Erie, United States (McKindles et al., 2021a). Sandusky Bay has a mean of 24°C during the cHAB season, so chytrid infection prevalence on *P. agardhii* was tested under laboratory conditions at temperatures ranging from 17.1°C–30.1°C and found the optimal temperature for infection was 21.7°C (McKindles et al., 2021b). Another study from cold water systems (average temperature of 8.47°C and 6.30°C) in Norway found also peak infection on *Planktothrix rubescens* at 21°C, which was the highest tested temperature (Rohrlack et al., 2015). While each of these study systems have different temperature ranges, they both had a similar infection thermal range, and both noted that there is a potential for thermal refuges to exist where *Planktothrix* can grow without any significant pressure from chytrid pathogenesis.

Chytrid zoospores are relatively small (2–6 μm) but have flagellated tails that can propel them through the water column in search of a host (Sime-Ngando, 2012). It has also been thought that chytrids seek out host through chemotaxis of chemicals released through photosynthesis. Phytoplankton release molecules creating a phycosphere to protect them or to aid in gathering resources (Bell and Mitchell, 1972). Reduced turbulence facilitates the development and establishment of the phycosphere, enabling the attraction of chemotropic organisms. In another host–parasite interaction, specifically the diatom (*Coscinodiscus granii*) and the parasitoid nanoflagellate (*Pirsonia diadema*), it was shown that water turbulence led to endemic infections, which effectively prevented the development of the host diatom (Kühn and Hofmann, 1999). They also noted that turbulent mixing could increase the chances of a parasite contacting the host, but that the contact time decreased along with that turbulent mixing (Kühn and Hofmann, 1999). *Planktothrix* and chytrid interactions have also been shown to have a negative relationship between turbulent mixing and infectivity (McKindles et al., 2021b). Additionally, *Planktothrix* can alter its buoyancy within the water column to allow optimal light and nutrient conditions. This ability to move throughout the water column coupled with water turbulence makes for an environment which chytrids may be unable to locate the hosts, despite being chemotactic.

In the light of global climate change there have been many studies focusing on the interplay of nutrient loading and temperature on the formation of cyanobacterial harmful algal blooms (cHABs). Fungal parasitism is an overlooked mechanism that can have effects on bloom food web dynamics, as well as control the diversity of cyanobacterial species (Gerphagnon et al., 2015). The goals of this study are to examine how environmental conditions can control and drive fungal parasitism in a natural setting, examining factors influencing endemic chytrid/*Planktothrix* interactions in a large, temperate freshwater reservoir.

## 2. Materials and methods

### 2.1. Large scale batch infection of Chytridiomycota on *Planktothrix agardhii*

#### 2.1.1. *Planktothrix* and chytrid maintenance and culturing conditions

To establish if Chytridiomycota infections on *Planktothrix agardhii* could be scaled up for mesocosm testing and to test the effects of water agitation on chytrid infection rates, preliminary experimentation was performed in lab in 15 L glass carboys. *P. agardhii* isolate 1,031 was grown as a unialgal, non-axenic batch culture (McKindles et al., 2021a). *Rhizophydiales* sp. isolate C02 were maintained on a variety of host strains to ensure no adaptations were developed before inoculation. Host and chytrid cultures were maintained in a Caron plant growth chamber (Marietta OH, United States), set at 22°C and 12:12 h light:dark cycles with cool fluorescent lights set to 15 μmol photons m^−2^ s^−1^. Cultures were maintained in Jaworski’s Medium (JM; Culture Collection of Algae and Protozoa).

#### 2.1.2. Large scale batch cultures

Two 15 L glass carboys were filled with 10 L of sterilized JM and set within a growth box that contained a halo bulb (Sylvania FP24/841/HO/ECO) that gives off approximately 100 μmol photons m^−2^ s^−1^ of cool white light. To lower the light intensity, a mesh filter was placed between the light and the carboy until the irradiance was approximately 10 μmol photons m^−2^ s^−1^ at the surface. The experiments occurred at room temperature (20°C–22°C). One carboy was kept stagnant, while the other had a small submersible water pump (Mountain Ark, Eagle UT, United States) attached to tubing to introduce water turbulence, which was estimated to have a circulation rate of 240 L h^−1^. Stagnant water carboy was treated as the control, as this experiment was testing the effect of water movement on infection on a large scale which previously had an inhibitory effect on infection prevalence in smaller lab cultures. *Planktothrix* was added to each carboy at an initial concentration of 100 filaments mL^−1^ which were allowed to grow and acclimate for 7 days. During this same time, the *Rhizophydiales* sp. isolate C02 (McKindles et al., 2021a) was infected on each host to familiarize the chytrid to its new host. After the week, the host was quantified as a measure of filaments mL^−1^ and the chytrid was quantified as a measure of infected filaments mL^−1^, where infected filaments contained one or more visible sporangia at either terminus (Gsell et al., 2013a; McKindles et al., 2021a). Additionally, to assess the densities of chytrids, sporangia per filament were counted and then used as a proxy for parasite density. The chytrid culture was added to the glass jar to reach an initial infection prevalence of 10% infected filaments.

Sample collection occurred right after chytrid introduction and continued approximately every 2 days for 20 days. Water samples were collected through a neoprene tube (Tygon S3 E-3603) to minimize disturbance of the culture and allowed for depth specific sampling, which included the settled population at the bottom of the jar and the suspended population toward the top of the vessel. Sample analysis included infection prevalence and both total and dissolved microcystin production, as described below.

### 2.2. Amplification of chytrid infection on *Planktothrix* sp. in mesocosms

#### 2.2.1. Grand Lake Saint Marys study site

**Figure 1:**
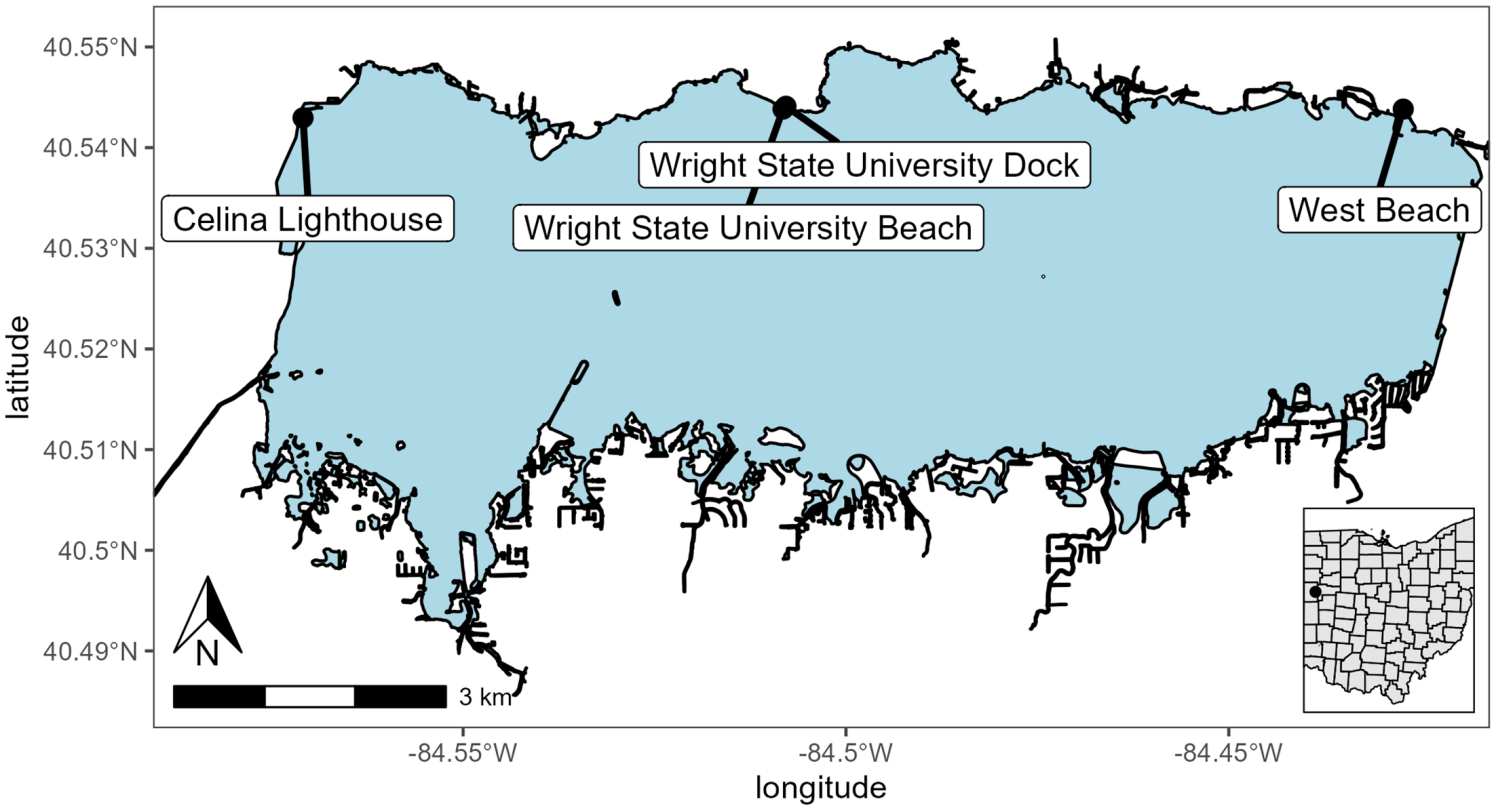
Sampling and mesocosm location for Grand Lake Saint Marys. The inset map shows the location of the lake in Ohio. Sites were Celina Lighthouse (40.54296079 and −84.57090855), Wright State University Beach and dock (40.54383316 and −84.50821996; 40.544115 and −84.507835, respectively), and west beach (40.54383316 and −84.42721725).

Grand Lake St. Marys (GLSM) is a reservoir located in western Ohio and is utilized for recreation, boating, fishing, and swimming. It has a mean depth of 1.6 m, a surface area of 5,000 ha, it is hypereutrophic, and has a long East to West wind fetch of 16 km (Figure 1; Welch et al., 2017). GLSM is known for its large blooms of *Anabaena, Aphanizomenon, Microcystis*, and *Planktothrix* with summer chlorophyll and total phosphorus (TP) averaging >100 μg L^−1^ (Hoorman et al., 2008; Filbrun et al., 2013; Steffen et al., 2014; Welch et al., 2017). Mesocosms were installed at the dock on the Lake Campus of Wright State University (lat/lon 40.543889, −84.507778). The dock is set into a manmade cove and was surrounded by the Wright State campus on the west side and the east side a farm field. The only access to the lake was from the south.

#### 2.2.2. Mesocosm design

A total of six mesocosms were installed off the dock. Each mesocosm is a 1.8 m × 28 cm (110 L volume) diameter clear polycarbonate cylinder open at the top and bottom and imbedded in the bottom sediment (Supplementary Figure 1). The mesocosms were filled with the natural community present in the water column at the start of the experiment. A cage was installed above the top opening to protect them from falling debris. The mesocosms were split into two treatments; three mesocosms had solar-powered water pumps to maintain water circulation at a rate of 240 L h^−1^, and the other three were left stagnant. Each week the mesocosms were assayed using a YSI EXO2 sonde to measure physiochemical parameters. Samples were taken from the top and bottom of each of the mesocosms for microscopy for infections, DNA extraction, chlorophyll extraction, microcystin concentration via ELISA, and nutrient analysis. After the sampling on 24 September 2021, the mesocosms were pulled from the sediment and replaced on the same site to allow for nutrient replenishment and to reset the physiochemical properties back to ambient fall weather lake conditions. This was done by lifting each of the mesocosms out of the sediment and allowing time for the sediment cloud to dissipate then resubmerging them into the water and setting them in the sediment. Additionally, samples were collected by Silvia Newell and Stephen Jacquemin in the open waters of the lake to provide a broader community analysis sites are indicated on Figure 1.

Samples for analysis were also collected outside of the mesocosms for use as a control to determine the impact the mesocosms were having on the general water quality parameters, and the measurements within each mesocosm were used to compare the differences between replicates and treatments. All measurements were taken both inside the mesocosms and immediately outside.

### 2.3. Sample processing and analysis

#### 2.3.1. Chytrid infection prevalence

Prevalence was calculated using a Sedgewick-Rafter counting cell. Each sample was counted under 100× magnification using the grids on the Sedgewick-Rafter as a reference. Samples were counted to 300 filaments or 15–1 μL grid squares with a minimum of 5 squares counted. Long filaments that were partially in the field of view were also counted by moving the microscope slide until the end of the filament was found. Prevalence was calculated by dividing the number of infected filaments over the total number of filaments inspected. Infection was determined by counting *P. agardhii* filaments with 1 or more sporangia attached to either terminus since infections only occur on the filament ends and infections are lethal (McKindles et al., 2021a,b). Prevalence of infection was reported as a percent infection of the total filaments mL^−1^.

#### 2.3.2. Particulate and dissolved microcystin toxin analysis

Microcystin samples were collected for both particulate and dissolved fractions during the duration of the experiment. The dissolved fraction was filtered through a 0.2 μm PTFE filter into glass vials and samples were frozen at −20°C until processed. The concentrations of particulate and dissolved microcystin toxins were measured using the Ohio EPA-approved Abraxis Microcystins-ADDA ELISA immunoassay (Abraxis LLC; Warminster, PA; EPA Method 546).

#### 2.3.3. DNA extraction, sequencing, and qPCR

DNA was taken at each time point at both the top (0.5 m) and bottom (1 m) depths. 200 mL of water was filtered through a 0.22 μm Sterivex filter (Millipore) on site and then stored in lab at −80°C until extraction. Samples from the 1 m depth at each time point were then extracted using a DNeasy PowerWater Kit (Qiagen, Germantown, MD, United States). Sample filter cartridges were cut open using a pipe cutter and the filter was removed prior to extraction per the manufacturer’s instructions. Each sample was quantified using a NanoDrop one microvolume UV-Vis spectrophotometer (Thermo Fisher Scientific, Waltham, MA, United States) to determine if the samples were appropriate for amplification and sequencing. Samples that had a concentration of at least 20 ng μL^−1^ were selected for further analysis and stored at −80°C. Samples were sent to HudsonAlpha Discovery Life Sciences (Hudson, AL, United States) for sequencing. Amplicon sequencing and QC was performed by HudsonAlpha Discovery Life Sciences using an Illumina MiSeq V3 with 600 cycle flow cells and up to 15Gb/25 M reads.

Real-time PCR was performed as outlined in McKindles et al. (2021b). In brief, the same volume of each extracted sample and 400 nM of each primer were run with PowerUp SYBR Green Master Mix (Thermo Fisher Scientific, Waltham, MA, United States) as described in McKindles et al. (2021b). Each sample was run under the same conditions multiple times using the different primer sets, as each reaction was a singleplex run. After an initial activation step at 50°C for 2 min and a denaturing step at 95°C for 2 min, 40 cycles were performed as follows: 15 s at 95°C, 30 s at 55°C and 60 s at 72°C. The efficiency of the rpoC1 primer set is 97.1% and the efficiency of the chytrid ITS primer set is 91.5% as described in McKindles et al. (2021b).

#### 2.3.4. Chlorophyll-a extraction

Chlorophyll-a was measured following filtration onto 0.2 μm polycarbonate membranes (Millipore) and extraction with Dimethyl sulfoxide (DMSO) at room temperature. After a 30 s sonification, each sample was left to extract overnight, followed by a centrifugation and measurement by fluorometry on a Turner TD-700 fluorometer (Turner Designs, Sunnyvale, CA) calibrated with a solid standard.

#### 2.3.5. Nutrient analysis

Nutrient samples were taken as whole water for total nutrients and filtered through a 0.22 μm Sterivex filter (Millipore) which was used as part of the DNA extraction protocol for the dissolved fraction. Both filtrate and whole water samples were sent for nutrient analysis. Nutrients were analyzed by analytical services on a SEAL AA3 autoanalyzer at The Ohio State University Stone Laboratory.

### 2.4. Molecular taxonomy

#### 2.4.1. 16S and 18S rRNA taxonomic assessment

Communities were analyzed targeting the 16S V3–V4 region and 18S V9 region. Amplicon sequence libraries were constructed using the dual index approach and the FASTQ files, along with the QC were then retrieved for further analysis.

Sequences from both 16S and 18S were processed using RStudio version 2022.07.2 working on R version 4.2.2 (2022-10-31). The DADA 2 pipeline (Callahan et al., 2016) was used for filtering, trimming, and taxonomic identification. Following DADA 2, sequences were added to the phyloseq package for further analysis. In short, samples were analyzed for quality of base calls for each sample in the forward and reverse directions. These QC scores were used to then trim sequencing to a QC threshold of 28. After trimming the primers and low-quality base calls, a DADA 2 error rate model was constructed then the sequences were dereplicated and finally the DADA 2 sequence error correction algorithm was applied to the sequences. These sequences were then merged using a 25-base pair and 12-base pair overlap with no mismatching bases for 16S and 18S, respectively. After merging, a sequence table was created, and chimeras were removed. Lastly, the sequence table was then assigned taxonomy using three separate sequence reference databases for 16S and one for 18S (Silva version 138.1, RDP trainset 16, GreenGenes version 13.8, and PR2 version 4.14.1). Following taxonomic assignment alpha diversity plots were constructed and relative abundance graphs were made to look at the community composition and relative abundance of taxa in each sample and treatment type. Finally, two beta diversity plots were constructed using Bray distances with both PCoA and NMDS methods.

### 2.5. Statistical analysis

Data analysis was done using RStudio version 2022.07.2 working on R version 4.2.2 (2022-10-31). Furthermore packages: Tidyverse (version 1.3.2), DADA2 (version 1.26.0), Vegan (version 2.6-4), phyloseq (version 1.42.0), and stats (version 4.2.2) were used for all data processing, statistical analysis, and graphing. Statistical significance was measured using two-way ANOVA and multiple pairwise comparisons were coupled with Tukey’s *post hoc*. Community relative abundances were compared using ADONIS and was corrected using the Benjamini Hochberg protocol.

## 3. Results

### 3.1. Large scale batch culture of chytrid infections on a toxin producing *Planktothrix agardhii*

**Figure 2:**
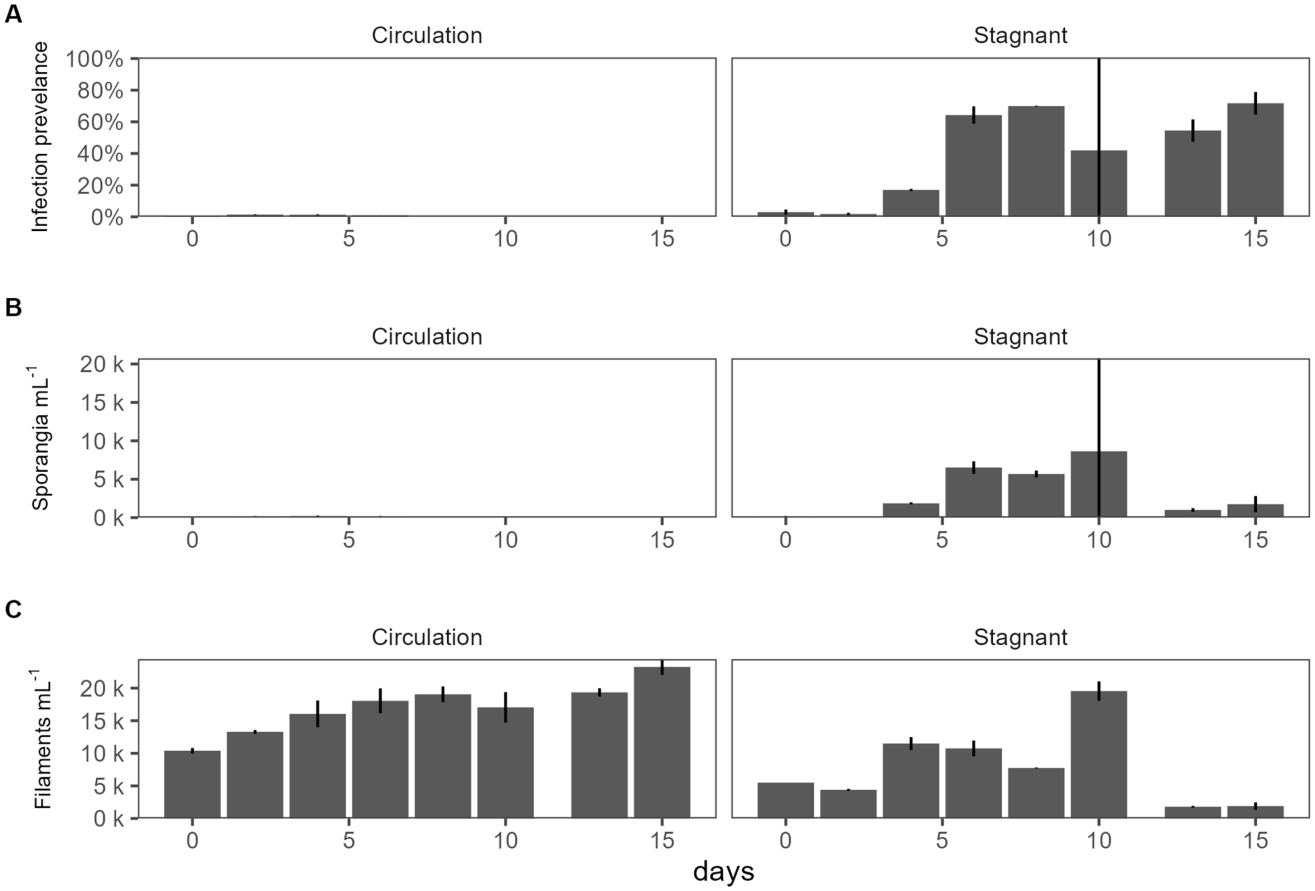
The effect of water circulation on chytrid isolate C2 pathogenesis on a toxin producing *Planktothrix agardhii* 1031. Batch culture experiment comparing water circulation and stagnant cultures. **(A)** Chytrid infection prevalence measured as percent of infected *Planktothrix* filaments over the total number of *Planktothrix agardhii* filaments. **(B)** Estimated number of sporangia. **(C)** Estimated number of total *Planktothrix agardhii* filaments. Error bars indicate standard deviation.

For the batch culture trial, a microcystin toxin-producing *Planktothrix agardhii* strain (strain designation 1,031) was used, and infected with chytrid isolate C2. A water pump was used to simulate natural water movement. The water circulation batch culture infection prevalence was 0–1.5% while the stagnant batch culture ranging from 1.20% to 83% (Figure 2A). Sporangia were counted as a proxy for chytrid abundances which were significantly different between the two cultures (*p* = 1.03E − 02; Figure 2B). There was no significant difference between the top and the bottom of the batch culture (Supplementary Figure 2). Infection prevalence were significantly different between the stagnant and the water circulation (*p* = 1.15E − 06; Supplementary Table 1). Because a microcystin producing *P. agardhii* strain was used, the dissolved fraction of toxin was tested to assess if chytrid infection increased toxin release in large cultures. Dissolved microcystin concentrations were consistently below 1 μg L^−1^ and concentrations between the water circulation and stagnant batch cultures were significantly different (*p* = 2.87E − 03; Supplementary Table 1). Looking at microcystin per filament of *Planktothrix* revealed differences between the two treatments (*p* = 1.05E − 08; Supplementary Table 1) and throughout the experiment (*p* = 2.47E − 06).

### 3.2. Physiochemical data for GLSM

**Figure 3:**
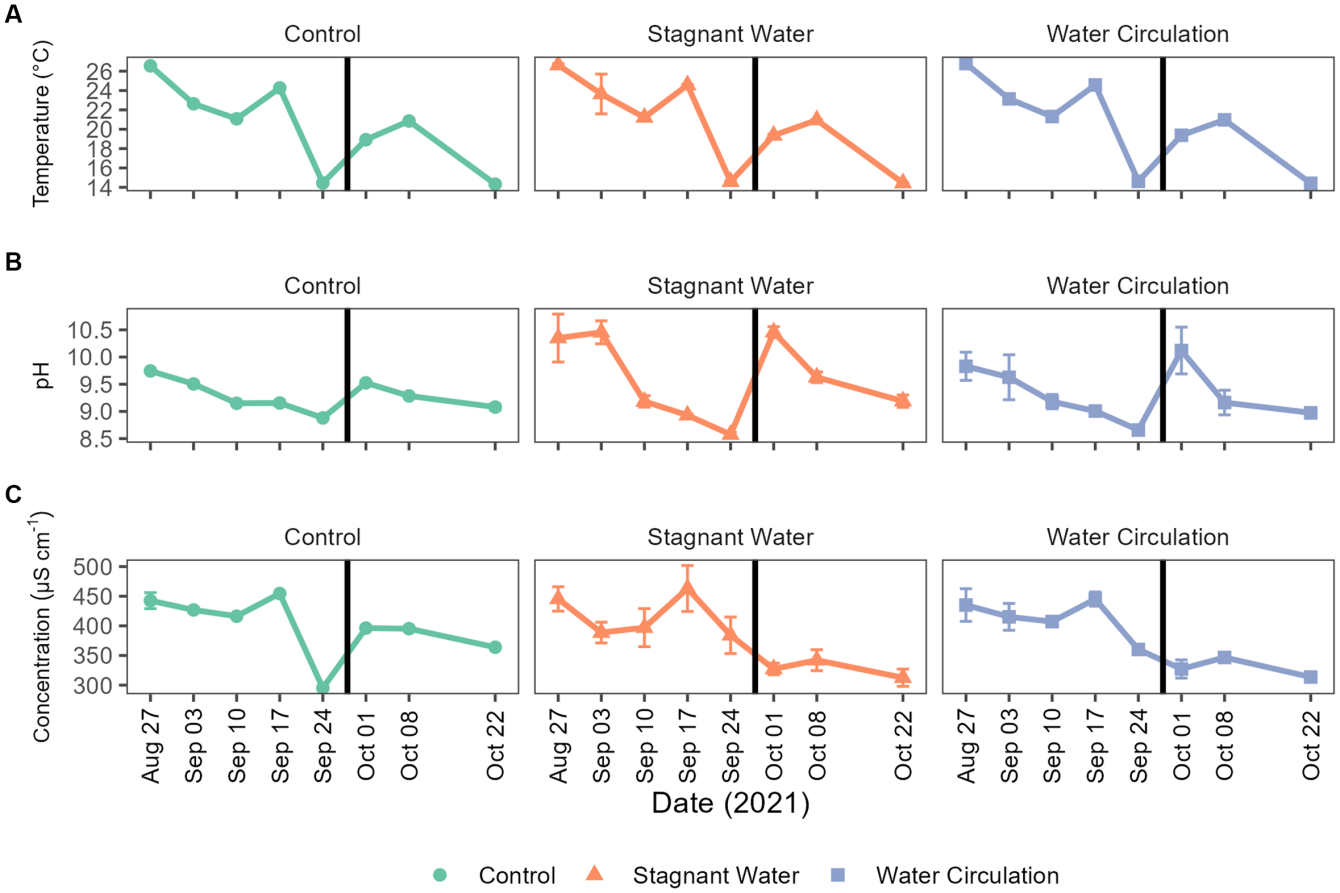
Abiotic parameters for Grand Lake Saint Marys and the mesocosms. **(A)** Temperature, **(B)** pH, and **(C)** conductivity measurements taken weekly. The black vertical line between 24 September 2021 and 01 October 2021 indicate the mesocosm reset point. Error bars indicate standard deviation.

To better understand the environment in which chytrid infections would occur, physiochemical data was collected within each mesocosm tube as well as in the cove waters for a period of 8 weeks, from 27 August 2021 to 22 October 2021. During this time, water temperatures ranged from 14.24°C to 27.83°C and had averages of 20.9°C ± 4.21°C, 20.7°C ± 4.24°C, and 20.7°C ± 4.18°C for control, stagnant, and circulation treatments, respectively (Figure 3A). Furthermore, over the 8-week period, temperatures were between 20°C and 22°C for about 25% of the time. The pH ranged 8.52–10.77 with stagnant water having the greatest fluctuations during the experiment (Figure 3B). Conductivity ranged 295.00–491.10 μS cm^−1^, again with the stagnant water mesocosms having the greatest range (Figure 3C). Lastly, Secchi disk measurements as a proxy for light attenuation had an average of 21 cm for all mesocosms during the experiment and ranged from 10 to 62 cm with the highest depth values taken in the water circulation mesocosms. During peak chlorophyll-a measurements, the stagnant water mesocosms developed a film of cyanobacteria, which decreased Secchi disk measurements for these samples (Supplementary Figure 4).

### 3.3. Nutrients

**Figure 4:**
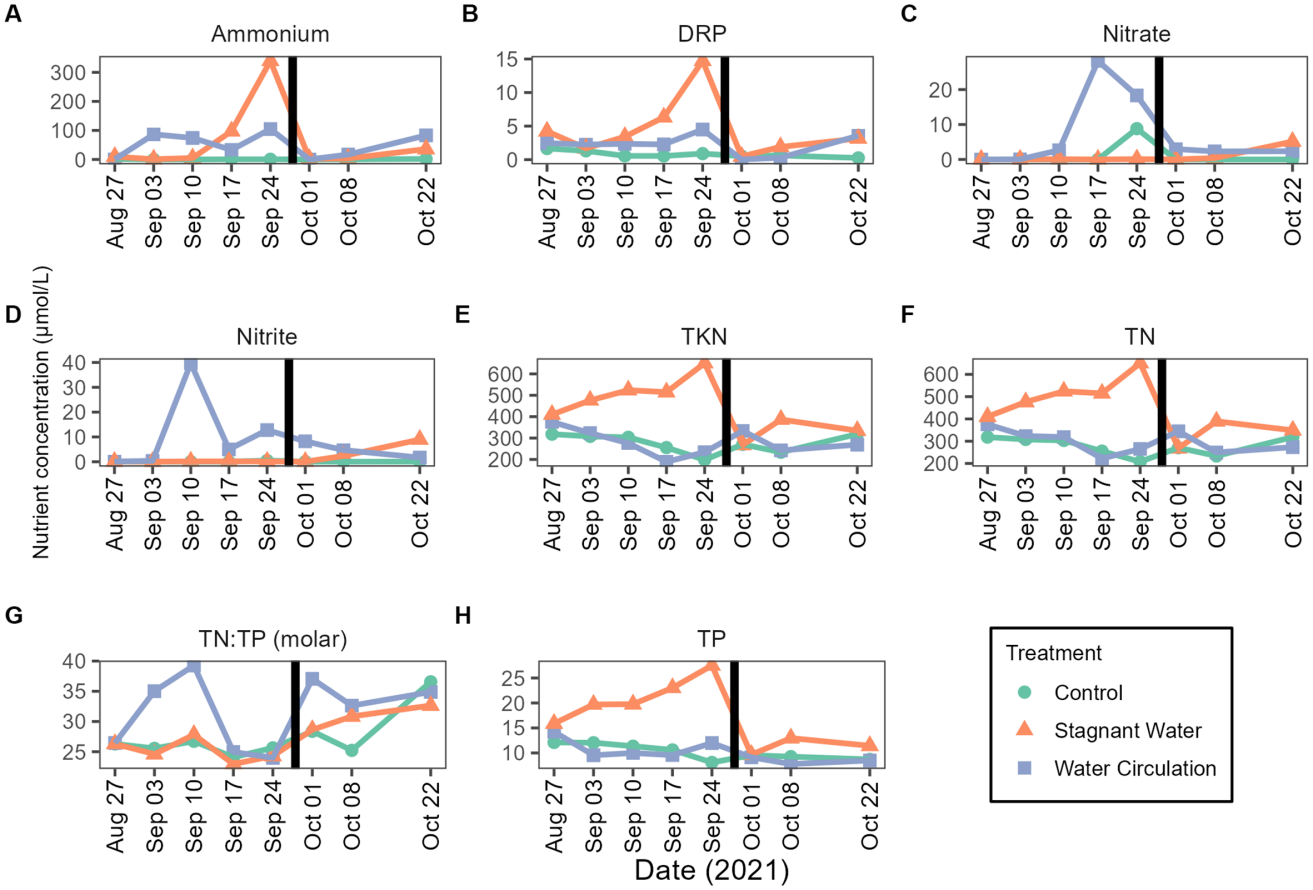
Mesocosms nutrient concentrations from 27 August 2021 to 22 October 2021. **(A)** Ammonium, **(B)** dissolved reactive phosphorus (DRP), **(C)** nitrate, **(D)** nitrite, **(E)** total kjeldahl nitrogen (TKN), **(F)** total nitrogen (TN), **(G)** TN:TP ratios as molar mass, and **(H)** total phosphorus (TP). The black vertical line between 24 September 2021 and 01 October 2021 indicate the mesocosm reset point. Please see Supplementary Table 3 for the complete data set (with standard deviations).

Nutrient samples were obtained throughout the experiment at the control site and in the mesocosms to track nutrient availability to the community. Nutrient concentrations during the experimental period were 4.09–33.05 μmol L^−1^ for total phosphorus and 138.24–694.16 μmol L^−1^ for total nitrogen (Figures 4H,F). Treatments had large differences between the replicate mesocosms causing high standard deviation (Supplementary Table 1). Interestingly, stagnant mesocosms had large spikes in ammonium, dissolved reactive phosphorus and total kjeldahl nitrogen (Figures 4A,B,E) while the water circulation treatment had large spikes of nitrate and nitrite early in the experiment (Figures 4C,D). These spikes could be caused by changes in pH and changes in dissolved oxygen (Supplementary Figure 5). Additionally, the spikes in the water circulation mesocosms could be caused by the circulation which could allow for nutrient resuspension. Total phosphorus and total nitrogen were significantly different between the treatments (*p* = 6.40E − 06 and *p* = 2.92E − 02 respectively). There were some differences between other individual nutrients during the experiment, see Supplementary Table 2. Furthermore, the spikes seen at date 24 September 2021, and leading up to that time correspond to a period when dissolved oxygen was low in both treatments with an average of 2.54 ± 1.36 mg L^−1^ and 1.60 ± 0.36 mg L^−1^ for stagnant and water circulation, respectively, on 24 September 2021 (Supplementary Table 3).

### 3.4. Chlorophyll-a analysis

**Figure 5:**
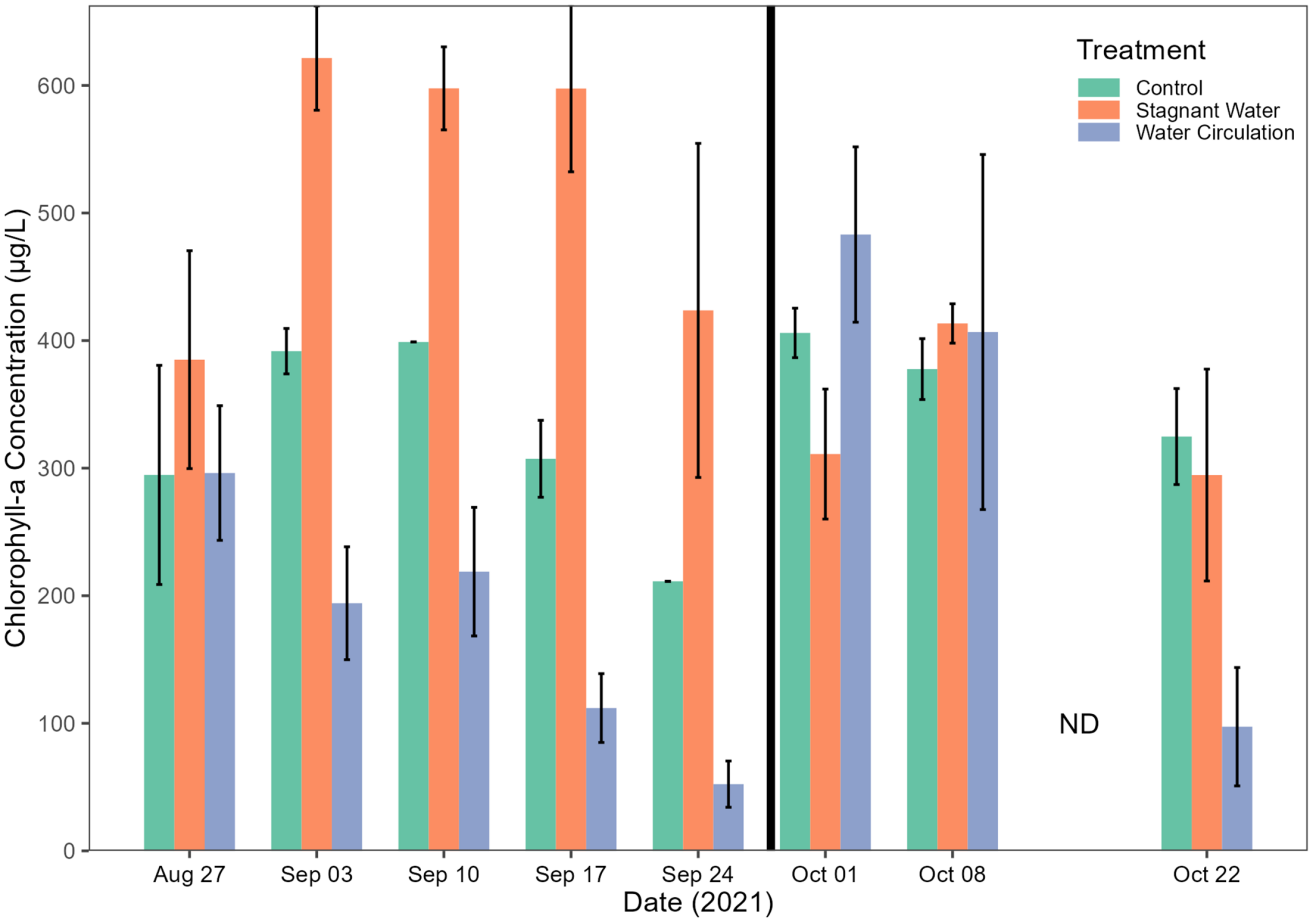
Chlorophyll-a concentrations. Error bars indicate standard deviation. The black vertical line between 24 September 2021 and 01 October 2021 indicate the mesocosm reset point.

Chlorophyll-a concentrations have been used as a proxy of productivity within a water body. Extractive chlorophyll concentration ranged from 24.77 to 896.5 μg L^−1^ and had an average of 356.6 ± 62.23 μg L^−1^, 376.5 ± 236.8 μg L^−1^, and 347.2 ± 304.9 μg/L L^−1^ for control, stagnant water, and water circulation, respectively (Figure 5). This high standard deviation is due to a large amount of variability between the six mesocosms and there was a cold spell where air temperatures dropped from 28.3°C to 13.4°C during the day and at night it went from 13.9°C to 7.8°C which caused the mesocosms to drop from an average of 24.53°C to 14.58°C across both mesocosm treatments and the control (Figure 3A). Stagnant mesocosm concentrations were significantly higher than the water circulation (*p* = 4.57E − 09) and the control treatment (*p* = 3.76E − 02) but water circulation was not significantly different from the control (*p* = 6.33E − 02; Supplementary Table 4). There was a noticeable decline in chlorophyll during the beginning part of the experiment, with an increase on 01 October 2021, when the mesocosms were reset, and a subsequent decline for the rest of the experiment until the mesocosms were removed on 22 October 2021.

### 3.5. Microcystin toxin concentrations

**Figure 6:**
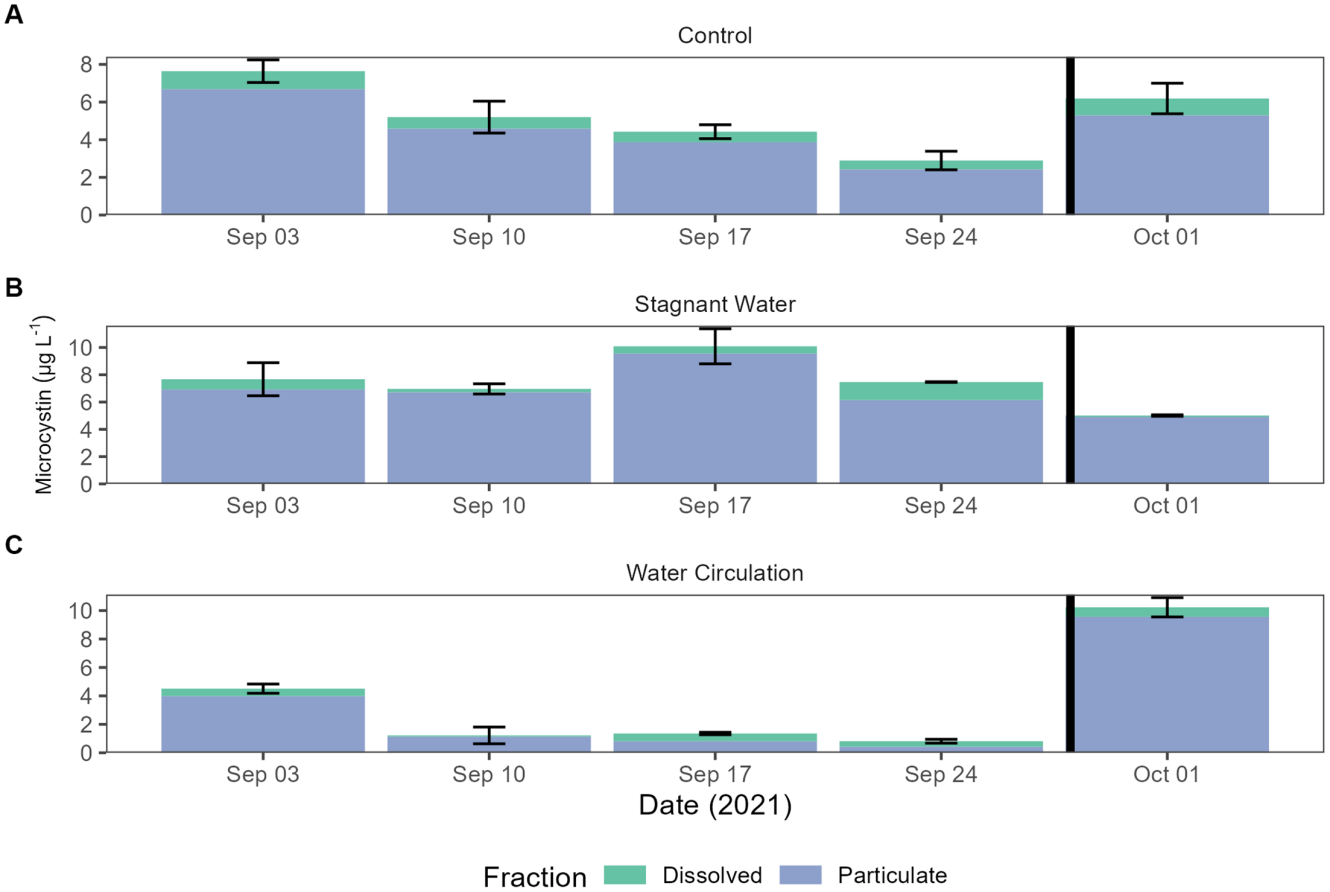
Microcystin concentrations for both total and dissolved. Error bars indicate standard deviation. **(A)** Control (cove water just outside mesocosm placement), **(B)** stagnant water mesocosms, and **(C)** water circulation mesocosms. The black vertical line between 24 September 2021 and 01 October 2021 indicate the mesocosm reset point.

Microcystin toxin concentrations were measured during the experiment to look for the potential increase in the dissolved fraction as a response to increasing cell breakage from chytrid infections. The World Health Organization (WHO) has set a recreation exposure guideline to 10 and 1.5 μg L^−1^ for drinking water. Both particulate and dissolved were measured by mixing the three mesocosms for each treatment into one sample for analysis. Dissolved concentration averaged 0.58 ± 0.32 μg L^−1^ and particulate concentrations averaged 4.87 ± 2.86 μg L^−1^. All samples from the particulate fraction were diluted 10-fold (Figure 6). Two-way ANOVA resulted in significant differences between all treatments and dates in particulate fraction (*p* = 7.79E − 08 and *p* = 5.90E − 06, respectively). The dissolved fraction was not significant between treatments and dates (*p* = 3.98E − 01 and *p* = 3.97E − 01, respectively). While particulate toxins were significantly different total toxins (both particulate and dissolved) was not significantly different between treatments and dates (Supplementary Table 5).

### 3.6. Microscopy infection counts

**Figure 7:**
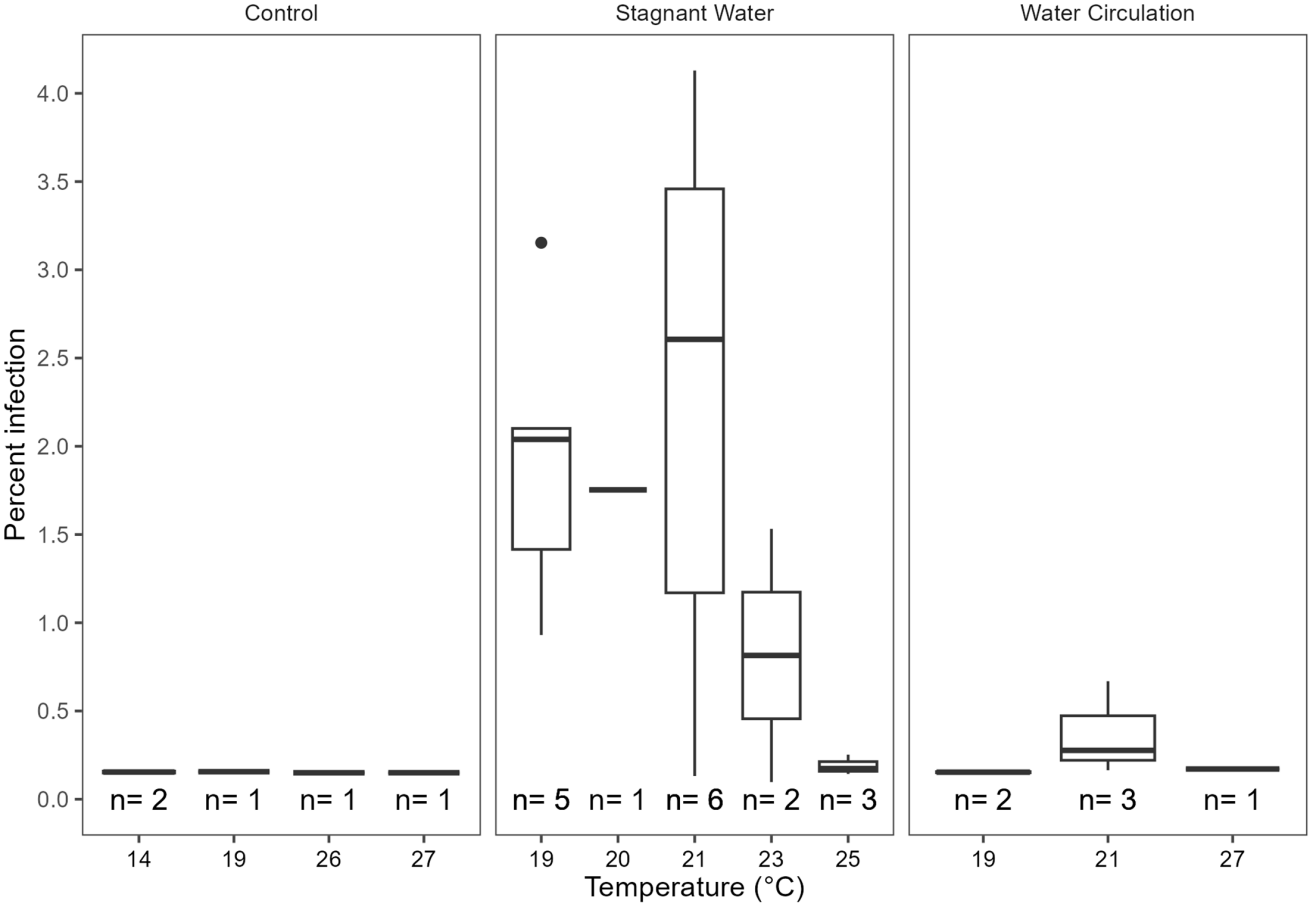
Calculated percent of infection at a given temperature separated by the treatment type control being the cove outside the mesocosms, stagnant the stagnant mesocosms, and the water circulation being the treatments with a water pump circulating water. The number of samples that had at least one identified chytrid infection within a temperature range are displayed as (*n*).

Infection prevalence was quantified using microscopy for the duration of the experiment. Infections reached a max of 4.12% infection with an average of 0.27% ± 0.76% over all samples and dates (Figure 7). There were infections in 25% of the samples examined (n = 112), with more infections occurring in the stagnant samples vs. the other two treatment types. There were trends within the data that suggest that 9.1–9.4 and 10.3–10.6 pH, 19°C–23°C, and 316–412 μS cm^−1^ were ideal for infections but due to the low number of samples with infections we could not statistically show significance. Furthermore, there was a significant difference between infections in the three treatment groups (*p* = 8.85E − 07, Supplementary Table 6) with the stagnant treatment being different from both the control (*p* = 1.05E − 03) and water circulation treatment (*p* = 1.72E − 06; Supplementary Table 7). There was no statistical difference in infections between the control and the water circulation treatment (*p* = 9.94E − 01). Temperatures between 19°C and 23°C had the highest percent of the counted filaments infected (Figure 7) and infections within this range were significantly higher than outside of the range (*p* = 1.52E − 05; Supplementary Table 8). Samples were taken at both top and bottom of each mesocosm and the control with no apparent difference between the two depths, as was seen as the *in vitro* experiments.

### 3.7. Community analysis

#### 3.7.1. 16S

**Figure 8:**
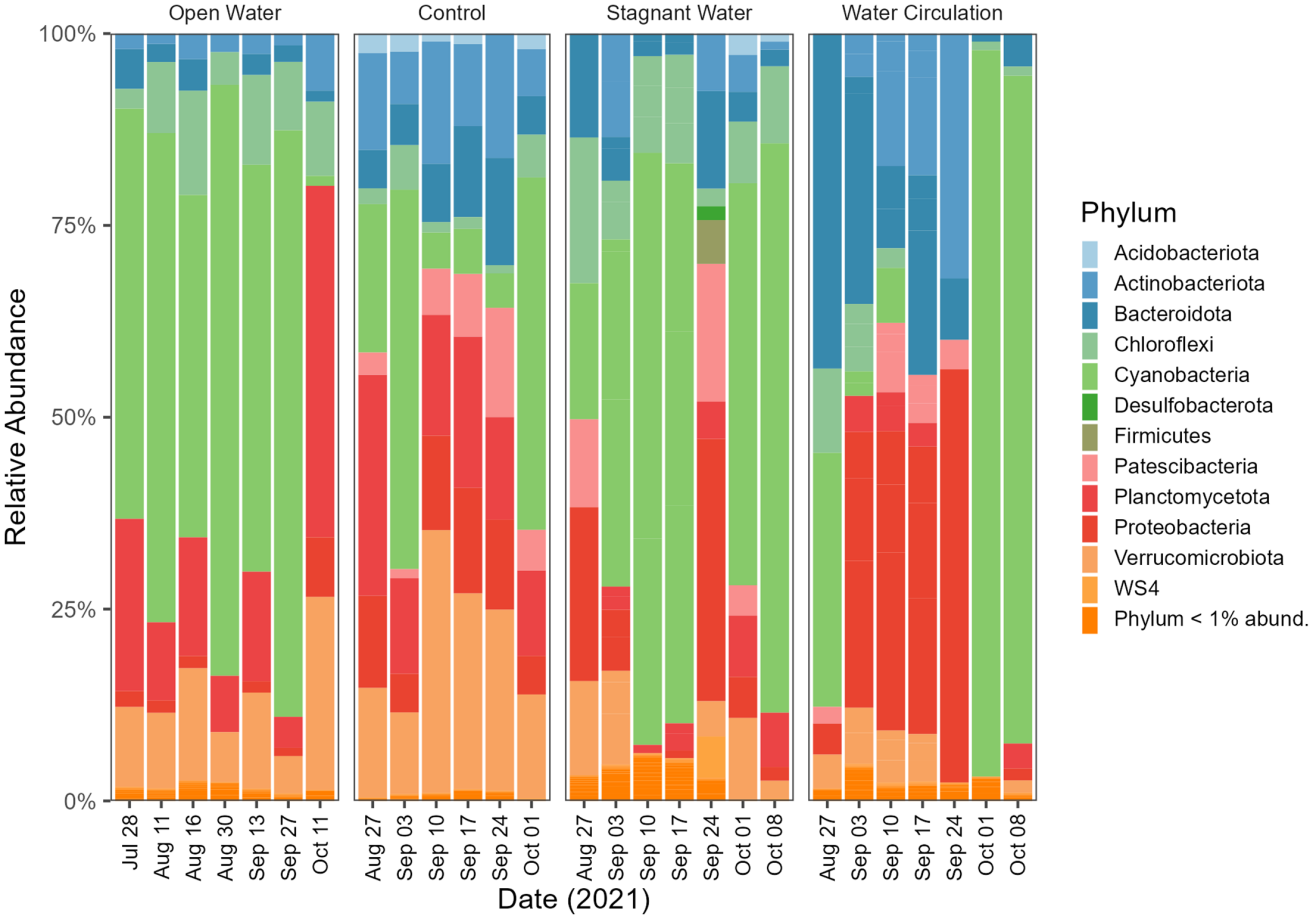
Bacterioplankton community composition at the phylum level. Open water is sites sampled outside of the cove where the mesocosms were installed. Control is within the cove next to the mesocosms. Stagnant water are mesocosms with no water circulation and the water circulation are mesocosms with a water pump moving water at 240 L h^−1^. Each treatment except for open water were sampled in triplicate and then pooled by date for community analysis.

In addition to the mesocosms, three sites (designated “open water”) were added along the north shore of GLSM for community analysis: West beach, Wright State University beach, and the lighthouse near the Celina water treatment plant intake (Figure 1). Phylum level relative abundance revealed that the stagnant water mesocosms and the open water sites were dominated by Cyanobacteria, apart from the Celina lighthouse (Figure 8). On the other hand, the water circulation mesocosms had less cyanobacteria over the course of this experiment and had higher abundances of Proteobacteria and Bacteroidota than all other treatments (Figure 8). Furthermore, the control for the mesocosms showed higher abundance of Verrucomicrobiota than the mesocosms, and less cyanobacteria than the open water and stagnant water mesocosms (Figure 8). Similar relative abundances and community composition were found between the water circulation treatment, control, and the Celina lighthouse. More specifically, the cyanobacteria were almost exclusively dominated by *Planktothrix agardhii* in all treatments including the open water samples. While many treatments were dominated by *Planktothrix agardhii* there were a few other cyanobacteria worth noting: *Cylindrospermopsis raciborskii* (1.2%–2.0%)*, Aphanizomenon gracile* (1.3%–7.4%), *Dolichospermum circinale* (1%–1.4%), and *Limnothrix* (1.3%; Supplementary Figure 6). Additional information regarding the alpha and beta diversities of the 16S and 18S community is in Supplementary material.

#### 3.7.2. 18S

**Figure 9:**
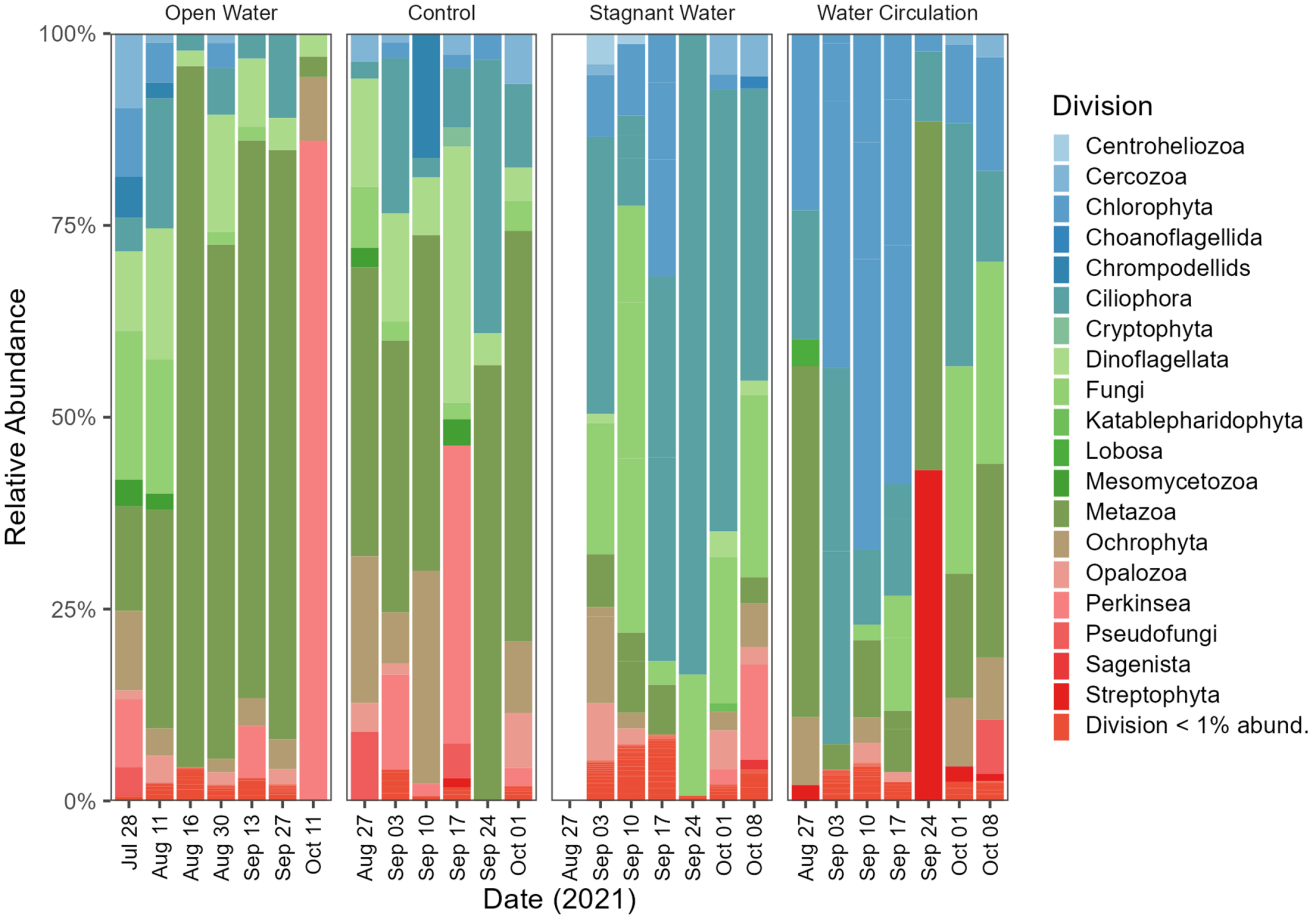
Eukaryotic community composition at the Division level. Open water is sites sampled outside of the cove where the mesocosms were installed. Control is within the cove next to the mesocosms. Stagnant water are mesocosms with no water circulation and the water circulation are mesocosms with a water pump moving water at 240 L h^−1^. Each treatment except for open water were sampled in triplicate and then pooled by date for community analysis.

The division level relative abundance data that was collected in this study highlights some differences between the eukaryotic communities of the different mesocosm treatments. Ciliophora and Fungi dominated the stagnant mesocosms with 8.8% and 12.4% relative abundance respectively, whereas Chlorophyta was more prevalent in the water circulation mesocosms with 14.9% relative abundance (Figure 9). The open water and control samples, on the other hand, were both dominated by Metazoa, with 19.7% and 8.5%, respectively (Figure 9). Furthermore, the specific genera of fungi that were identified in the control, open water, and stagnant mesocosms provide additional insight into the microbial communities present in each treatment. The domination of *Rhizidium*, *Rhizophydium*, and *Rhyzophidiales_X* (Supplementary Figure 7) in the control, open water, and stagnant mesocosms, respectively, could be due to the high abundance of *P. agardhii* as these genera have several species known to be parasitic on the cyanobacterial host. Additionally, there are several groups of ciliates (Ciliophora) that are known to consume *P. agardhii* including the species *Urotricha, Vorticella* and *Coleps* (Supplementary Figure 8). All three of the taxa listed here were found in abundances between 1% and 70% of the total ciliate abundances.

#### 3.7.3. Quantitative PCR

**Figure 10:**
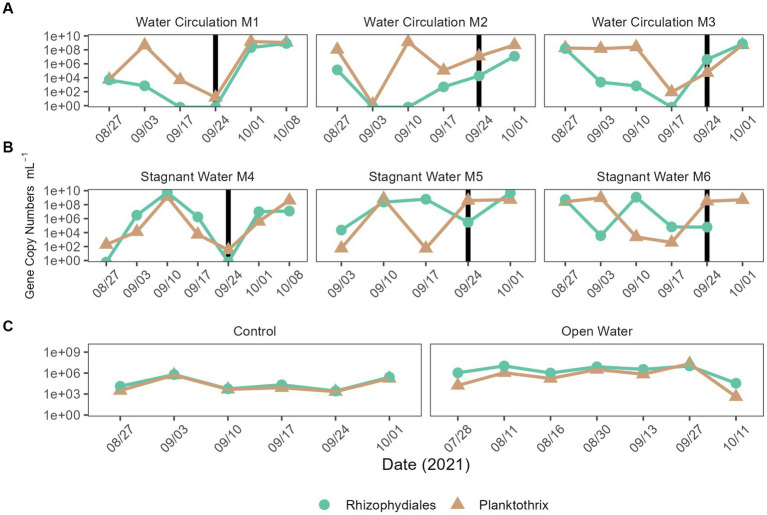
qPCR quantification of *Planktothrix agardhii* and the *Planktothrix*-specific chytrid. **(A)** Water circulation mesocosm treatments, **(B)** stagnant water mesocosm treatments, and **(C)** are the controls. The control group consists of two groups the “Control” which is right outside the mesocosm, and “Open Water” are from sites within the lake but outside of the cove where the experiment took place. Vertical lines represent the date where the mesocosms were reset after sampling.

Quantitative polymerase chain reaction (qPCR) was used to confirm microscopic trends in the abundances of chytrids (*Rhizophydiales*) and *Planktothrix* during this experiment. A total of 100 samples were tested for the genetic signatures for both *Planktothrix* and *Rhizophydiales*; of those samples, 86% of them detected *Rhizophydiales* and 100% of them detected *Planktothrix* (Figures 10A–C). Regardless of treatment, both chytrid and *Planktothrix* abundances mirrored each other with some occasional lag time (Figures 10A–C). There was statistical difference in abundances of chytrid (*p* = 2.99E − 02) *Planktothrix* genetic signatures (*p* = 9.54E − 03) between all the treatments including the open water samples (Supplementary Table 9). Pairwise analysis showed that *Planktothrix* was statistically different in the mesocosms vs. the control samples (Supplementary Table 10), but *Planktothrix* abundances between the mesocosm treatments were not significantly different (Supplementary Table 10). Chytrid abundances were only significantly different between the water circulation and stagnant water treatments (*p* = 3.65E − 02; Supplementary Table 10).

## 4. Discussion

### 4.1. Large scale batch culture experiments

*In vitro* batch cultures were utilized to help predict the dynamics of *in situ* mesocosm experiments by controlling for all factors except for water agitation. Agitation using a water pump maintained similar levels of biomass between the stagnant and water circulation carboy and indicated that water movement could significantly reduce chytrid infections on *P. agardhii* (Figure 2). Additionally, *Planktothrix* growth under moving water and stagnant water conditions are different. In batch culture water movement increased the growth rate of *Planktothrix*. This could be due to the introduction of CO_2_ from the movement on the surface layer of water or from the lack of parasite infectability. This confirmed previous work that suggested water agitation would reduce chytrid infections in the wild, either by preventing zoospores from finding their host through signal disruption or by preventing swarming and attachment (McKindles et al., 2021a,b).

The presence of microcystin, a toxin of great concern, was also evaluated, as it was previously suggested that chytrid infection could cause the cells to release high concentrations of microcystin into the dissolved fraction. However, the batch culture experiment showed that even when the chytrid infection prevalence reached 40%–60%, there was almost no detectable dissolved microcystin presence (0.06 to 0.6 μg L^−1^). Therefore, chytrid infections may not have a significant impact on microcystin production by *Planktothrix*, which support data in another recent study (Agha et al., 2022).

### 4.2. Grand Lake St. Marys mesocosms

The main goal of this work was to build upon previous studies looking at the environmental effects on chytrid infection on *Planktothrix agardhii* within the context of a real environment. The mesocosms were accessing an environment without amendment. Most studies have been conducted in laboratory-controlled conditions, which are important to get an estimation of what is possible, but *in situ* studies like the one conducted here can confirm lab observations and identify other potential factors that may constrain or maximize chytrid pathogenesis. Our 16S and 18S community analysis showed a population shift following installation of the mesocosms. GLSM has a dominant *Planktothrix* bloom through many months of the year (Steffen et al., 2014), meaning it would be an ideal study location.

Physiochemical measurements during the sampling period indicated that both temperature and conductivity were well suited for chytrid pathogenesis as found in previous studies (McKindles et al., 2021b). In lab studies using local to Ohio isolates of both host and parasite indicated that infections were most prominent at 0.226–0.426 mS cm^−1^ (McKindles et al., 2021b), which was consistent with the conductivity range of our study site at GLSM (0.246–0.491 mS cm^−1^; Figure 3). Temperature was also indicated to have a significant effect on infection prevalence, with an optimal infection temperature of 21°C (McKindles et al., 2021b). Similarly, our mesocosm had the highest precent of infections between 19°C and 22°C (Figure 7). During the sampling period, the mesocosm were exposed to a wide range of water temperatures, from 14°C to 28°C (Figure 3). Infections could occasionally be identified at these extreme temperatures, but they were infrequent and not at a high prevalence (Figure 7), further suggesting the significance of thermal refuges for chytrid infections. Previous work looking at infection dynamics of Chytridiomycota and *Planktothrix* species has identified these thermal refuges in which the host is able to grow at temperatures which are limiting to the parasite. In controlled laboratory studies, Rohrlack et al. (2015) identified a lower thermal refuge for *Planktothrix rubescens* at temperatures below 11.6°C, while McKindles et al. (2021b) identified an upper thermal refuge for *Planktothrix agardhii* at temperatures above 27.1°C. To date many of the experiments looking into chytrid-host interactions have been laboratory based and have reported infection rates that are not seen in the environment. More real-world experimentation like what is being done here should be done to elucidate what factors are preventing chytrid pathogenesis in the environment.

Nutrients in Grand Lake Saint Marys have been an ongoing topic of concern (Jacquemin et al., 2018). During the 8 weeks that the mesocosms were in total nitrogen in the control ranged from 138.24–694.16 μmol L^−1^ (Figure 4F) and the total phosphorus ranged from 4.091–33.05 μmol L^−1^ (Figure 4H). In the water circulation mesocosms, there was a spike of nitrate (Figures 4C,D) causing a high TN:TP ratio which could have allowed for *Planktotrhix* to be less competitive (Figure 4G). These spikes on 20 September 2021 and leading up to that time correspond to a period when dissolved oxygen was low in both treatments (Supplementary Figure 5). One possible explanation for the increase could be the decreasing temperature or the large rain event that dropped nearly 12.7 cm of rain, reducing phytoplankton production and thus relieving the use of these nutrients causing the spike. Interestingly, stagnant mesocosms had large spikes in ammonium, dissolved reactive phosphorus and total kjeldahl nitrogen (Figures 4A,B,E) this was also during the time when temperatures were low and right after the rain event. Ammonium plays a key role in sustaining *Planktothrix* blooms (Hampel et al., 2019), despite this, chlorophyll-a declined on 24 September 2021 and did not fully recover.

By reducing the water flow and creating a stagnant environment with the untreated mesocosm, both the bioactivity and cyanobacterial toxin production increased. Measurements of chlorophyll-a were used a proxy for bioactivity for each treatment, with significantly higher values in the stagnant water mesocosms compared to the water outside of the mesocosms, which was higher still than the water circulation mesocosm (Figure 5). The increased chlorophyll-a measurements for the stagnant water mesocosms were due to an increase in abundance of cyanobacteria, particularly *Planktothrix* species (Figure 8; Supplementary Figure 6). Due to *Planktothrix* being a fast migrating buoyant cyanobacteria, it is able to take advantage of stable, stagnant water column by adjusting its depth for optimal light conditions (Carey et al., 2012). This increase was also reflected in the increases in microcystin total toxin concentrations (Figure 6). In the batch culture experiment, the stagnant water treatment had a reduced *Planktothrix* carrying capacity, whereas in the mesocosms, *Planktothrix* was more prevalent in the stagnant condition than the water circulation treatments despite having more parasite pressures. This is likely because in the environment the *Planktothrix* community is more diverse than a single strain that is known to be susceptible to infection like in the batch culture. In other studies where they look at mixed populations of both host and parasite they find that mixed cultures leads to a decline in infection prevalence (McKindles et al., 2023). While McKindles et al. utilized smaller benchtop experiments more lab experiments with mixed cultures would likely be necessary to see if non-susceptible strains of *Planktothrix* present in the stagnant water of the batch culture could then thrive as seen in the environmental mesocosms. While the cyanobacterial population dominated in the stagnant water mesocosms, it was replaced by a Chlorophyta population in the water circulation mesocosm (Figure 8; Supplementary Figure 11), perhaps indicating other environmental factors are involved population shifts besides parasitic pressure.

Within the stagnant water mesocosms, *Planktothrix* proliferated and so did the organisms that could lead to their demise. 18S reads indicated that the stagnant mesocosm was dominated by Ciliophora, Dinoflagellata, and Fungi. Ciliates are an important group of microorganisms in freshwater ecosystems, playing key roles in controlling algal populations and nutrient cycling and can therefore play an important role in mitigating the negative impacts of algal blooms. Many of the taxa within the Ciliophora and Fungi division were ones that have been reported to consume (Filip et al., 2012; Combes et al., 2013; Farthing et al., 2021) or parasitize *Planktothrix* species (Kagami et al., 2007; Agha et al., 2016; Frenken et al., 2017; McKindles et al., 2021a,b). Combined with the measures of diversity provided by the 16S and 18S data, this study reveals the importance of water movement in structuring the phytoplankton population. Overall, water movement suppresses *Planktothrix* dominance, despite the reduction of chytrid parasitism by coexisting *Rhizophydiales*. Combined with the fact that infections were rarest at 23°C and 25°C, the effects of climate change and lake warming may further reduce chytrid success. Consequently, the role of chytrid pathogenesis has limited impact in this system, despite the potential importance of trophic upgrading to grazers in the food web (Gerphagnon et al., 2019).

## 5. Conclusion

Fungal parasitism is an overlooked mechanism that can have effects on bloom food web dynamics, as well as control the diversity and relative abundances of cyanobacterial species. Chytrid pathogenesis reached a maximum of 4% with infections mostly occurring in the stagnant mesocosm. Temperature coupled with water movement factors limiting or restricting chytrid pathogenesis in temperate lakes. As more systems are receiving anthropogenic nutrient additions, understanding the mechanisms that control bloom severity is of great importance as chytrid pathogenesis also may play an important role in trophic upgrading and nutrient recycling within the ecosystem.

## Data availability statement

The datasets presented in this study can be found in online repositories. The names of the repository/repositories and accession number(s) can be found at: https://www.ncbi.nlm.nih.gov/, PRJNA950135.

## Author contributions

RW: conceptualization and writing—original draft preparation. RW and KM: methodology, formal analysis, and investigation. RW, KM, and GB: writing—review and editing and supervision. GB: funding acquisition and resources. All authors contributed to the article and approved the submitted version.

## Funding

This work was supported by funding from the National Institutes of Health (1P01ES028939-01) and the National Science Foundation (OCE-1840715) awards to the Bowling Green State University Great Lakes Center for Fresh Waters and Human Health.

## Conflict of interest

The authors declare that the research was conducted in the absence of any commercial or financial relationships that could be construed as a potential conflict of interest.

## Publisher’s note

All claims expressed in this article are solely those of the authors and do not necessarily represent those of their affiliated organizations, or those of the publisher, the editors and the reviewers. Any product that may be evaluated in this article, or claim that may be made by its manufacturer, is not guaranteed or endorsed by the publisher.
