# Supplementary material for: Effects of water movement and temperature on Rhizophydium infection of Planktothrix in a shallow hypereutrophic lake
Source: Front Microbiol. 2023 Jun 19;14:1197394. doi: 10.3389/fmicb.2023.1197394 (PMC10345987; doi:10.3389/fmicb.2023.1197394)
Supplement: Supplementary file 1 [file Data_Sheet_1.pdf]

Supplementary Material

Effects of Water Movement and Temperature on *Rhizophydium* Infection of *Planktothrix* in a Shallow Hypereutrophic Lake

Ryan S. Wagner[1,2], Katelyn M. McKindles[2,3], George S. Bullerjahn[1,2]*

[1]Department of Biology, Bowling Green State University, Bowling Green, Ohio, USA

[2] Great Lakes Center for Fresh Waters and Human Health, Bowling Green State University, Bowling Green, Ohio, USA

[3] Ecology and Evolutionary Biology, College of Literature, Science, and the Arts, University of Michigan, Ypsilanti, Michigan, USA

*** Correspondence:** George Bullerjahn: bullerj@bgsu.edu

1 Supplementary Data

1.1 16S and 18S community diversity

Alpha diversity analysis of the 16S rRNA data revealed relatively low variation in richness across the four treatments (Supplemental Figure 9A). The observed number of taxa varied between treatments, with stagnant water exhibiting the lowest average diversity, followed by the open water and water circulation, and the control having the highest diversity. Notably, the stagnant treatments showed the highest evenness across samples, except for one outlier (mesocosm 4 on September 24, 2021). To ensure that the measurement was not limited by sampling effort, all alpha diversity tests were conducted with and without rarefaction, and the results were consistent (Supplemental Figure 10). The Shannon diversity index showed similar trends as the observed measurement, except for species evenness. Further statistical analysis using two-way ANOVA and Tukey's HSD test revealed a significant difference between stagnant water and the control ($p = 2.42E-03$) and between water circulation and the control ($p = 1.96E-02$) but not between water circulation and the stagnant water (Supplemental Table 11). Beta diversity analysis of the samples indicated that there were significant differences in the communities based on the different treatment types ($p = 1.00E-05$) and dates ($p = 2.97E-02$; Supplemental Figure 11; Supplemental Table 12).

Alpha diversity analysis of the 18S rRNA data yielded similar results to those of the 16S community, with more variation observed in evenness among the sample treatments (Supplemental Figure 9B). However, ANOVA and Tukey's HSD test showed no significant differences in the communities (Supplemental Table 13), and the rarefied and non-rarefied data produced similar results. Overall, the Shannon diversity index showed that all the sample treatments were composed of similar communities, however there was some differences in species evenness likely because of the smaller, rarer communities.

2 Supplementary Figures and Tables

2.1 Supplementary Figures

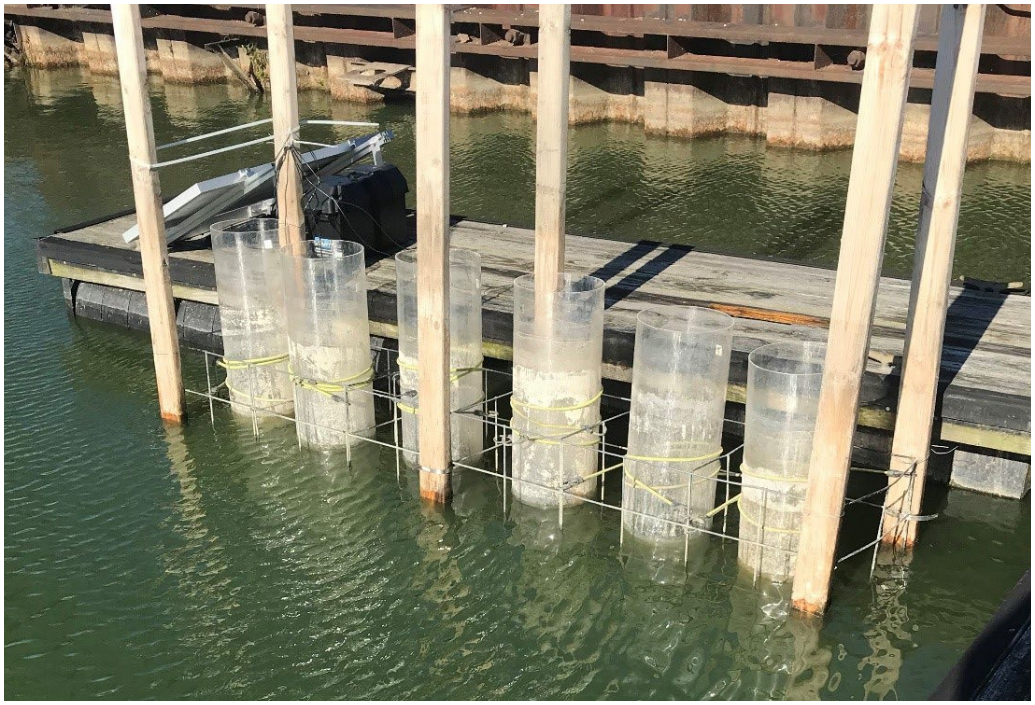

Supplemental Figure 1. Grand Lake Saint Marys mesocosm installation on the dock. Three tubes were water circulation, and 3 tubes were stagnant water.

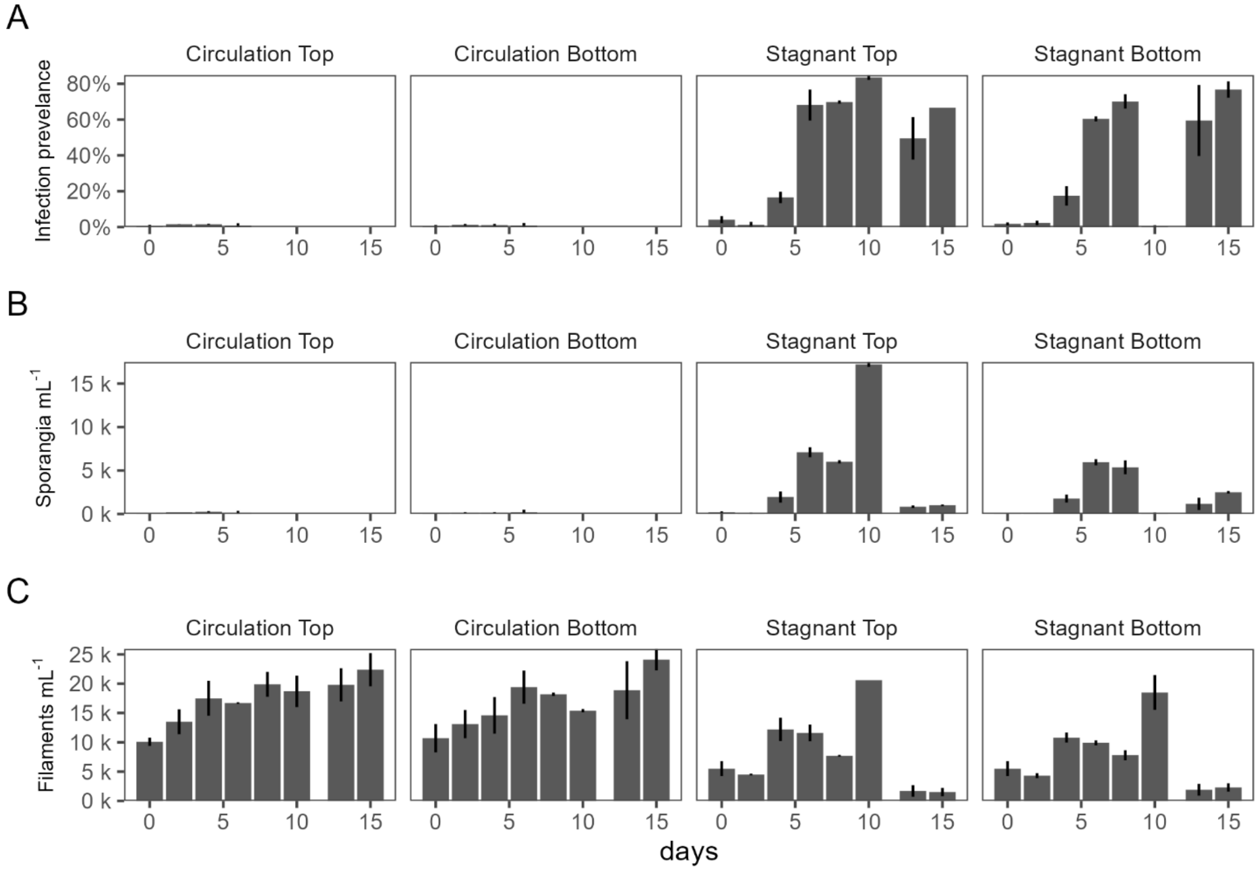

Supplemental Figure 2. The effect of water circulation and depth on chytrid isolate C2 pathogenesis on a toxin producing Planktothrix strain 1031. Batch culture experiment comparing circulation and stagnant cultures. (A) Chytrid infection prevalence measured as percent of the total number of healthy non-infected Planktothrix filaments. (B) Estimated number of sporangia or vegetative chytrid cells. (C) Estimated number of Planktothrix filaments. Error bars indicate standard deviation.

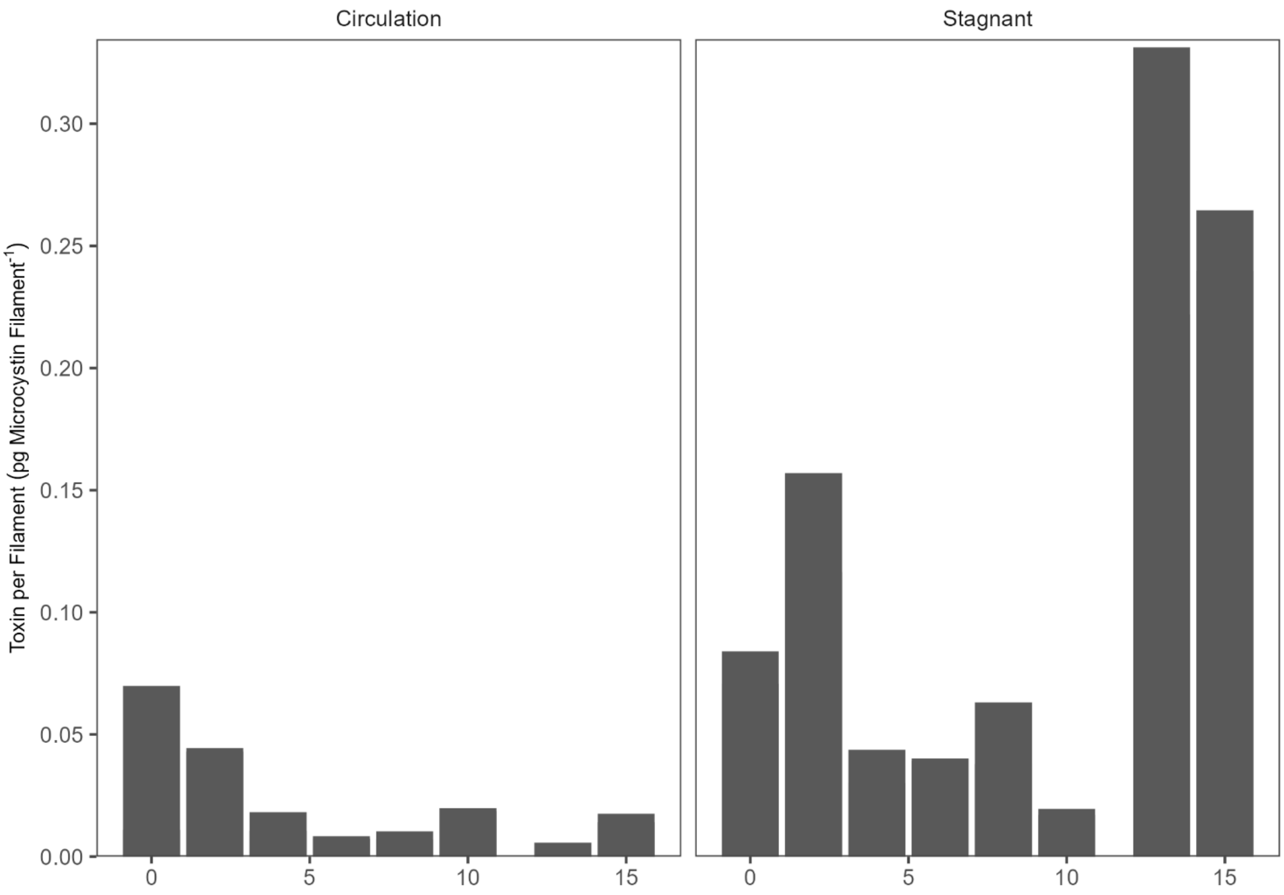

Supplemental Figure 3. Large batch culture microcystin concentrations. Toxins were measured in the dissolved fraction and taken as microcystin concentration per filament.

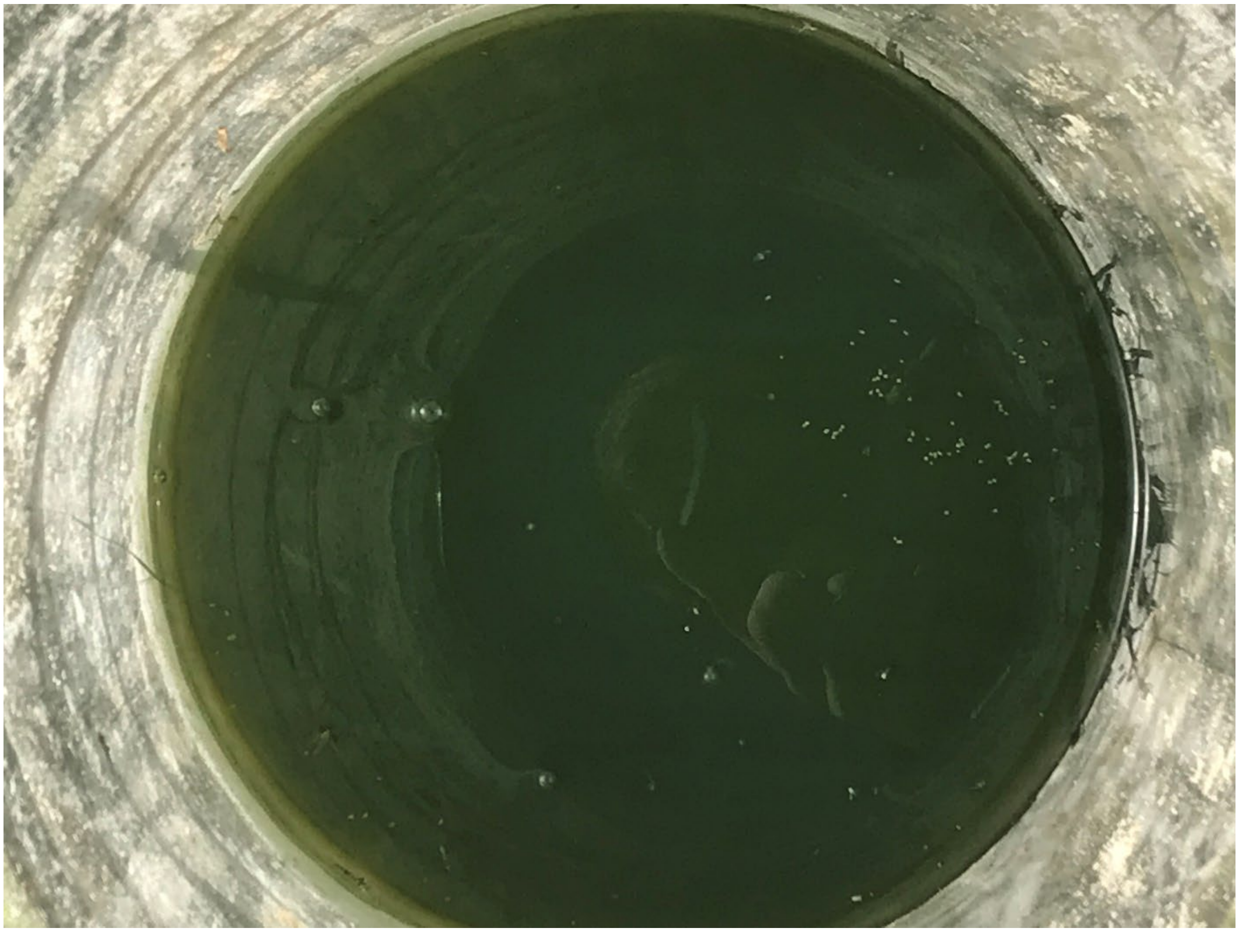

Supplemental Figure 4. Inside film of the stagnant mesocosm at Grand Lake Saint Marys.

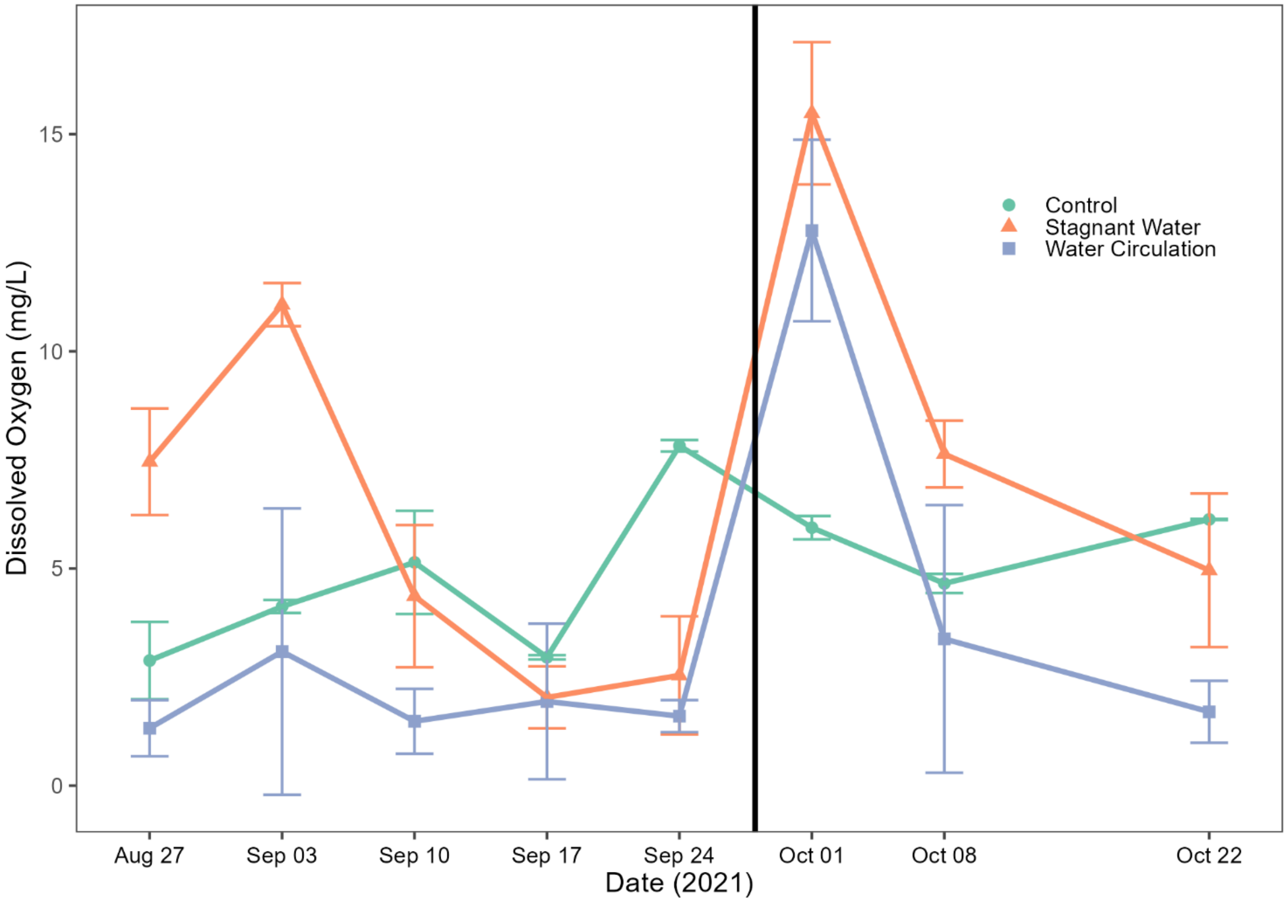

Supplemental Figure 5. Dissolved oxygen measurements for Grand Lake Saint Marys Wright State University's dock and the two mesocosm treatments. The black vertical line represents the time where the mesocosms were reset by lifting them out of the water and setting them back in.

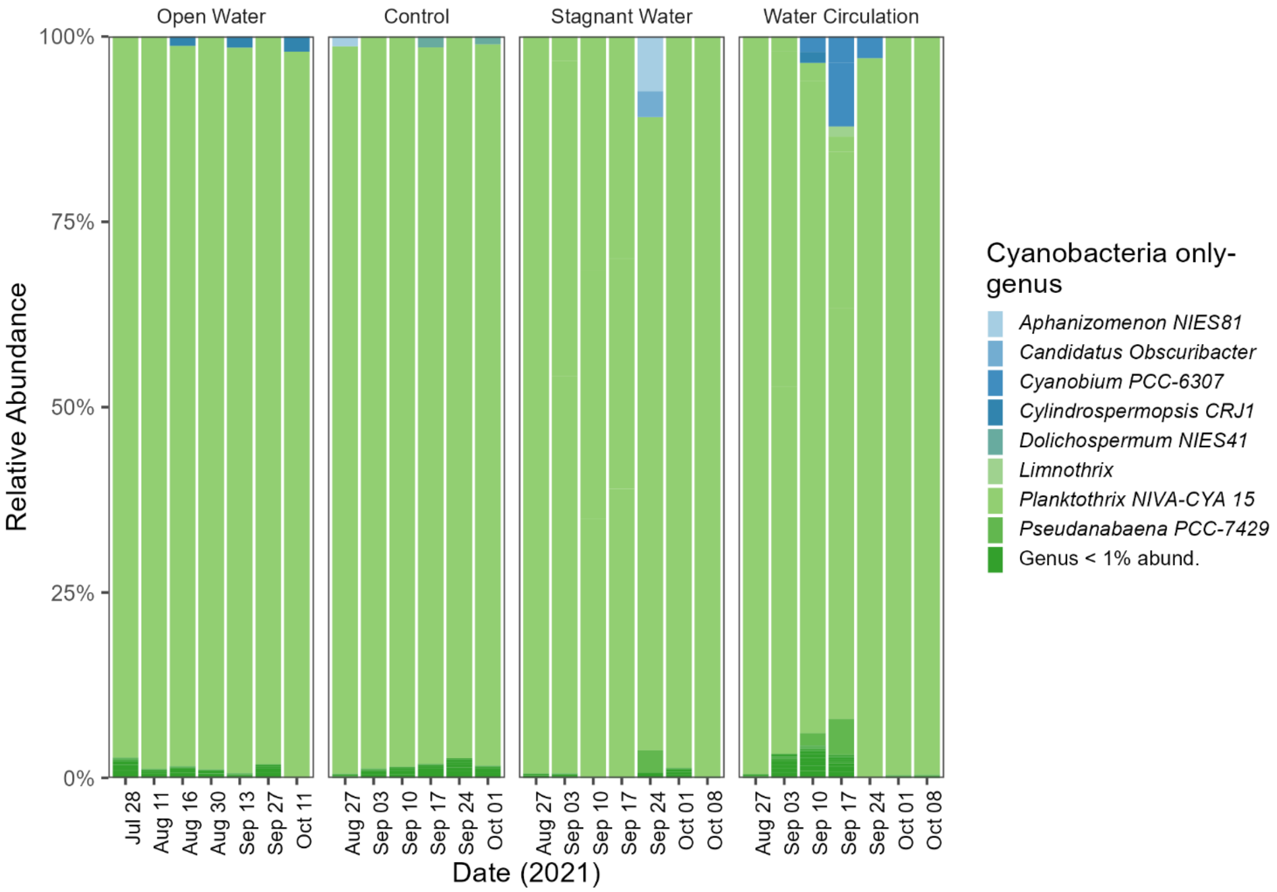

Supplemental Figure 6. Bacterioplankton community composition within the phylum Cyanobacteria at the genus level. Open water refers to sites sampled outside of the cove where the mesocosms were installed. Control is within the cove next to the mesocosms. Stagnant water are mesocosms with no water circulation and the water circulation are mesocosms with a water pump moving water at 240 L h^{-1}. Each treatment, except for open water, were sampled in triplicate and then pooled by date for community analysis.

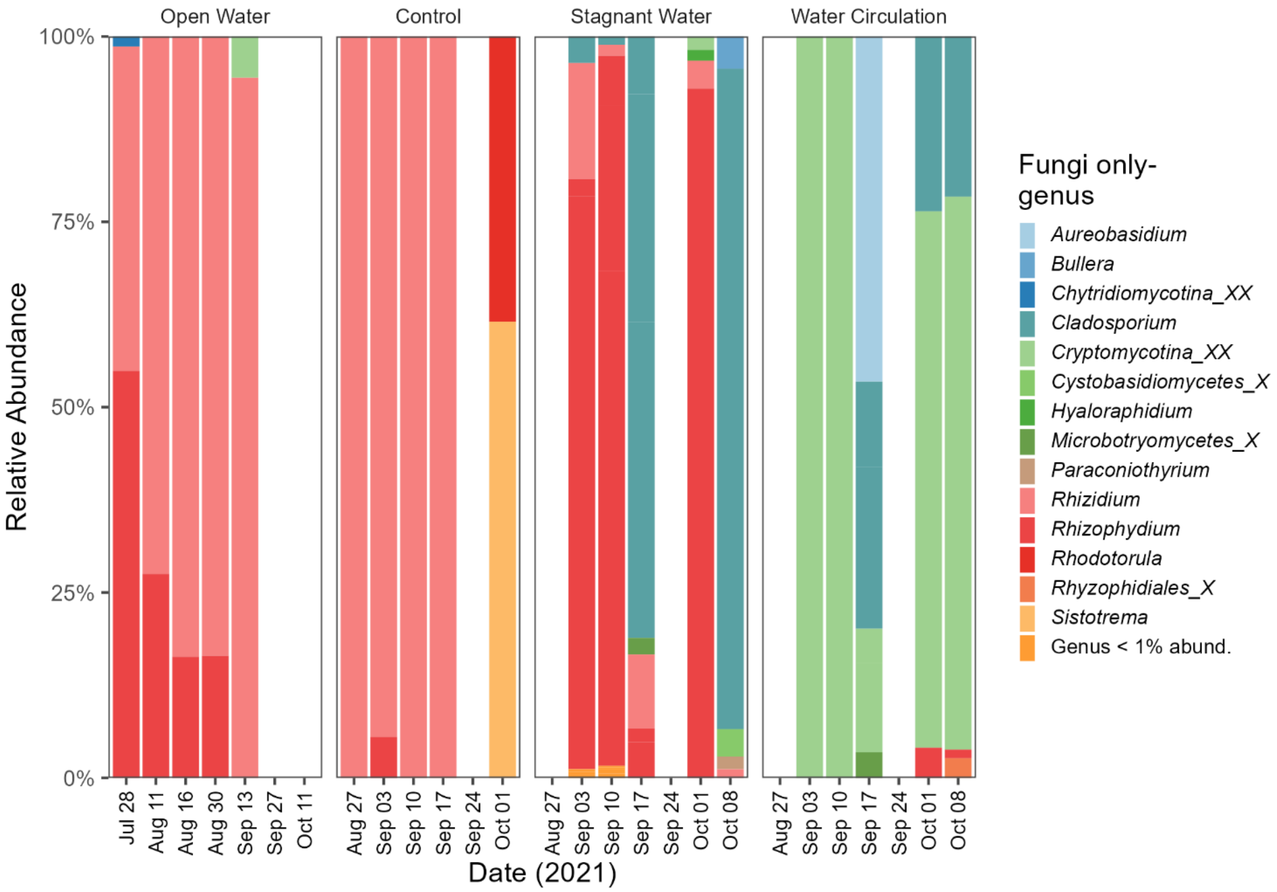

Supplemental Figure 7. Eukaryotic community composition of the Fungi at the genus level. Open water refers to sites sampled outside of the cove where the mesocosms were installed. Control is within the cove next to the mesocosms. Stagnant water are mesocosms with no water circulation and the water circulation are mesocosms with a water pump moving water at 240 L h^{-1}. Each treatment except for open water were sampled in triplicate and then pooled by date for community analysis.

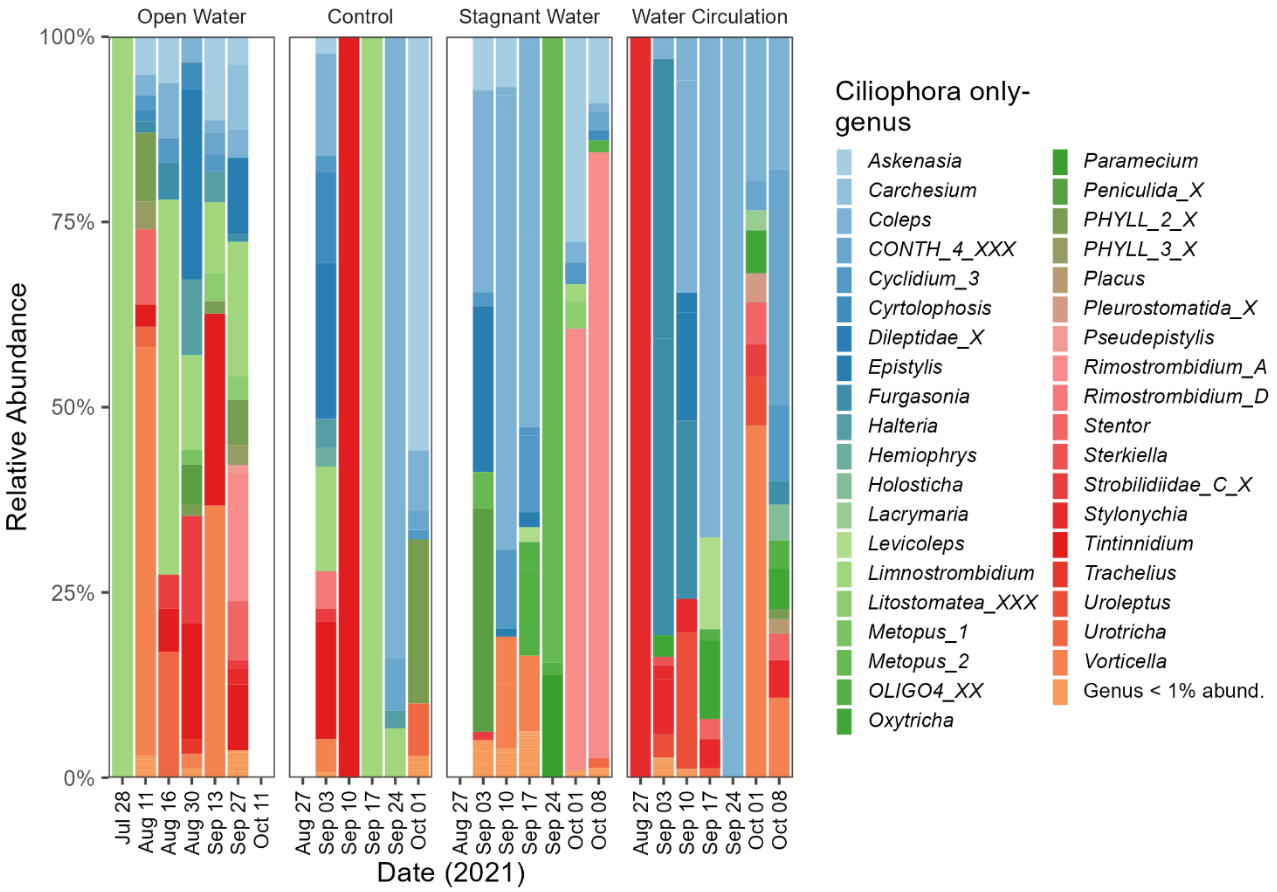

Supplemental Figure 8. Eukaryotic community composition of the Ciliophora at the genus level. Open water refers to sites sampled outside of the cove where the mesocosms were installed. Control is within the cove next to the mesocosms. Stagnant water are mesocosms with no water circulation and the water circulation are mesocosms with a water pump moving water at 240 L h^{-1}. Each treatment except for open water were sampled in triplicate and then pooled by date for community analysis.

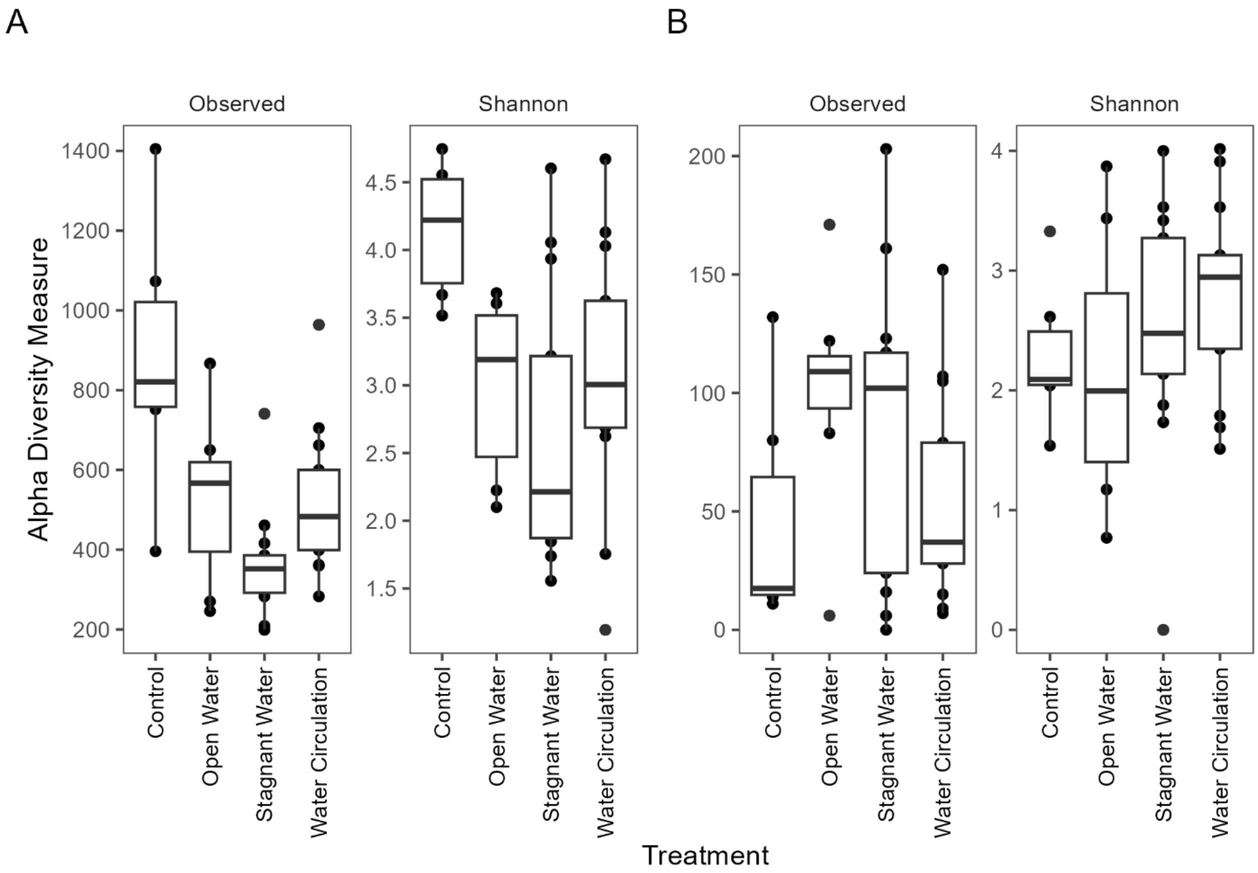

Supplemental Figure 9. Boxplots of alpha diversity measures for both bacteria and eukaryote communities. (A) Bacterial, (B) Eukaryotic community alpha diversity with the observed values with the Shannon index. Both plots use unrarefied data and are grouped by treatments.

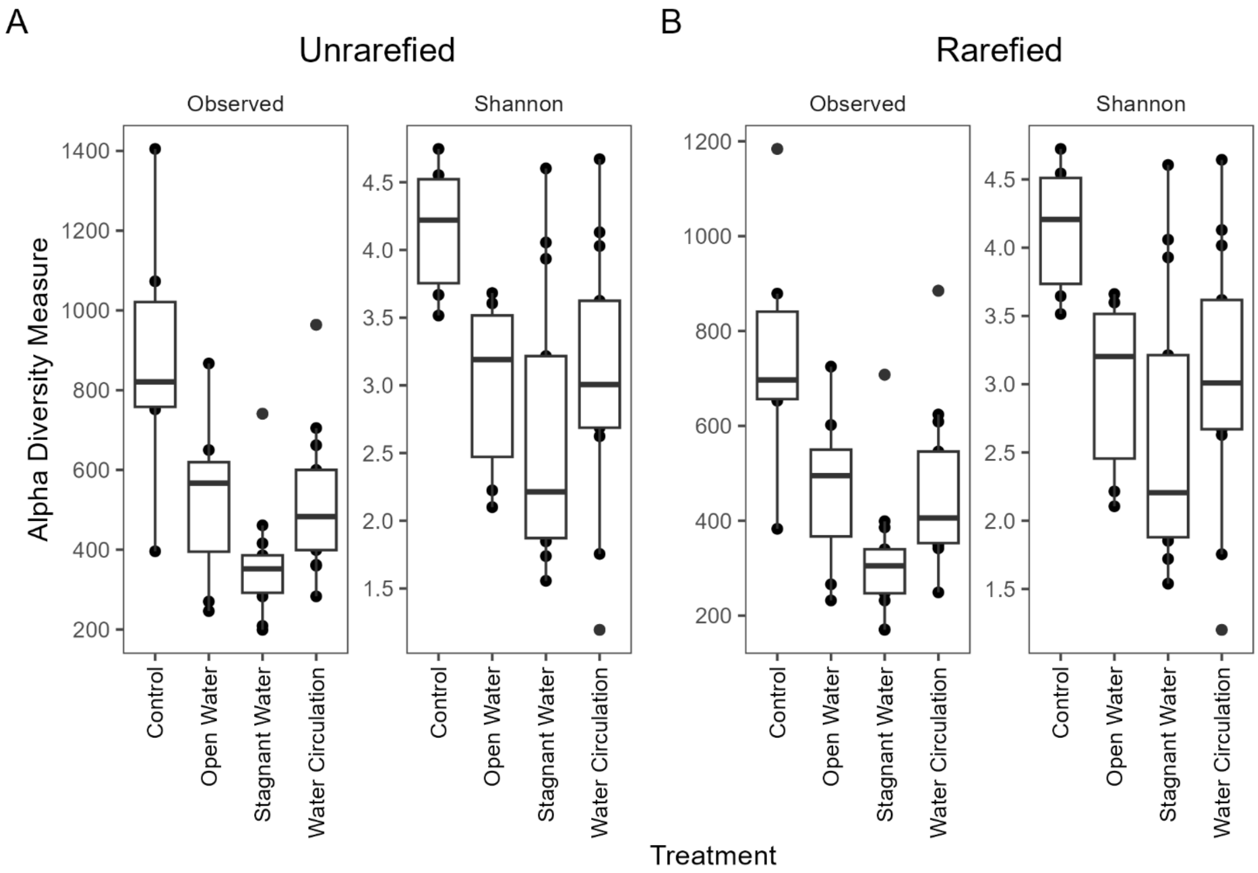

Supplemental Figure 10. Boxplot of alpha diversity for both unrarefied and rarefied data. (A) Unrarefied, (B) rarefied data with alpha diversity measurement for the observed and Shannon index.

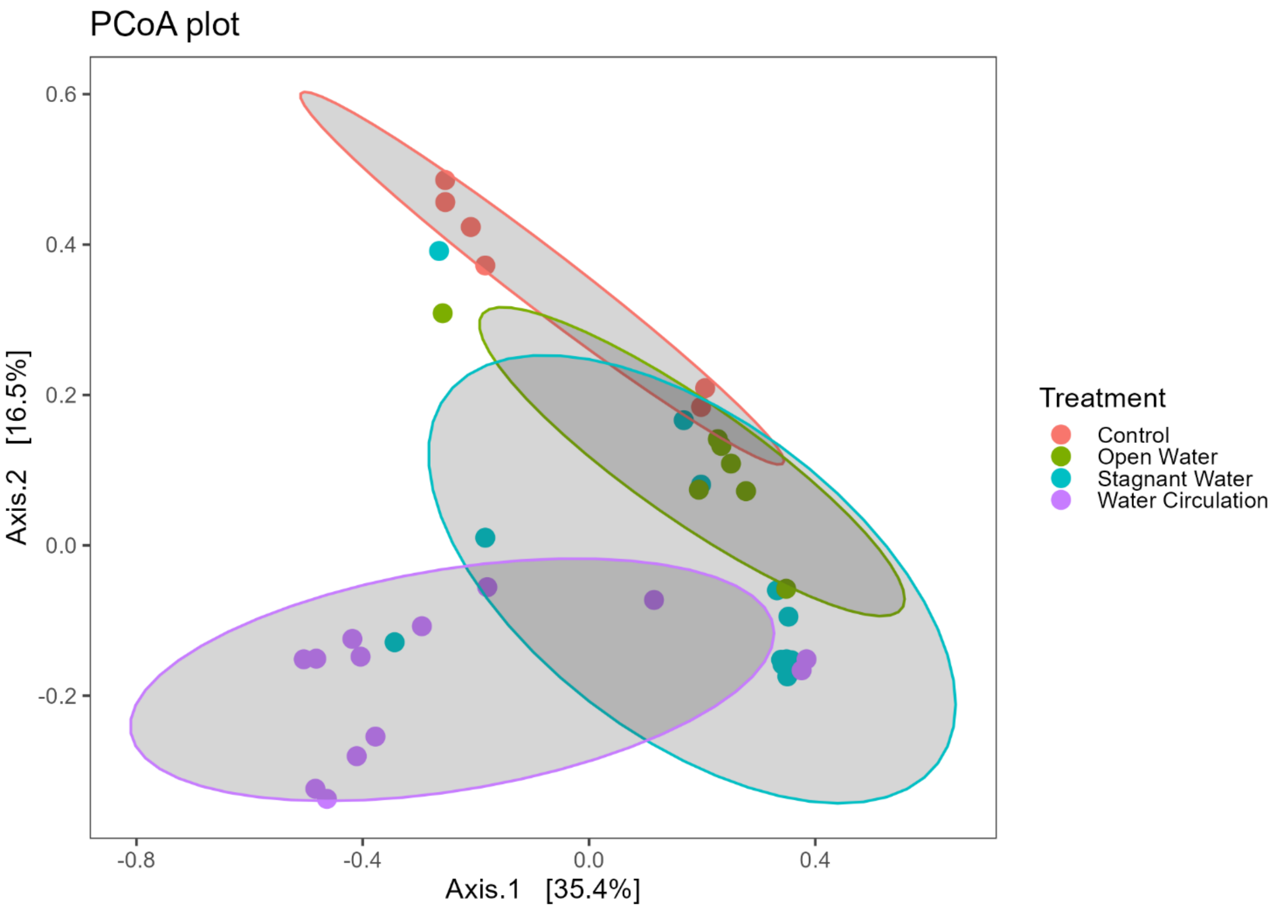

Supplemental Figure 11. Principal coordinate analysis (PCoA) plot showing the community Bray-Curtis distances of each sample. Ellipses were drawn to better display the grouping for each treatment.

2.2 Supplementary Tables

Batch culture results						
Measurement	Comparisons	DF	SS	MS	F stat	P
Infection prevelance	Flow	1	1.28e04	1.28e04	5.71e01	**1.15e−06**
Infection prevelance	Date	7	5.91e03	8.45e02	3.77e00	**1.32e−02**
Infection prevelance	Flow:Date	7	6.23e03	8.91e02	3.98e00	**1.06e−02**
Sporangia per mL	Flow	1	7.87e07	7.87e07	8.46e00	**1.03e−02**
Sporangia per mL	Date	7	7.38e07	1.05e07	1.13e00	3.91e−01
Sporangia per mL	Flow:Date	7	7.54e07	1.08e07	1.16e00	3.78e−01
Filaments per mL	Flow	1	6.73e08	6.73e08	4.93e02	**1.91e−13**
Filaments per mL	Date	7	3.10e08	4.42e07	3.24e01	**2.71e−08**
Filaments per mL	Flow:Date	7	4.02e08	5.75e07	4.21e01	**3.90e−09**
Microcystin per filament	Flow	1	6.75e−02	6.75e−02	1.15e02	**1.05e−08**
Microcystin per filament	Date	7	7.07e−02	1.01e−02	1.71e01	**2.47e−06**
Microcystin per filament	Flow:Date	7	7.64e−02	1.09e−02	1.85e01	**1.45e−06**
Microcystin concentrations	Flow	1	2.15e−01	2.15e−01	1.24e01	**2.87e−03**
Microcystin concentrations	Date	7	3.25e−01	4.64e−02	2.67e00	**4.92e−02**
Microcystin concentrations	Flow:Date	7	1.60e−01	2.29e−02	1.32e00	3.04e−01
Bold indicate significance. All statistics are run as two-way ANOVA.						

Supplemental Table 1. All statistical results from the batch culture experiment. Bold indicates significance and all measurements were run as two-way ANOVA with interactions.

Mesocosm nutrient results						
Measurement	Comparisons	DF	SS	MS	F stat	P
Ammonium	treatment	2	2.51e04	1.25e04	2.60e00	9.23e−02
Ammonium	Date	6	1.69e05	2.82e04	5.84e00	**4.80e−04**
Ammonium	treatment:Date	12	1.37e05	1.14e04	2.37e00	**2.96e−02**
Nitrite	treatment	2	1.00e03	5.01e02	1.40e00	2.62e−01
Nitrite	Date	6	1.28e03	2.14e02	6.00e−01	7.28e−01
Nitrite	treatment:Date	12	2.23e03	1.86e02	5.22e−01	8.82e−01
DRP	treatment	2	1.01e02	5.04e01	4.10e00	**2.74e−02**
DRP	Date	6	2.94e02	4.90e01	3.99e00	**5.19e−03**
DRP	treatment:Date	12	1.78e02	1.48e01	1.21e00	3.25e−01
Nitrate	treatment	2	6.21e02	3.11e02	1.25e00	3.01e−01
Nitrate	Date	6	9.24e02	1.54e02	6.21e−01	7.12e−01
Nitrate	treatment:Date	12	1.30e03	1.08e02	4.36e−01	9.35e−01
TP	treatment	2	7.90e02	3.95e02	1.89e01	**6.40e−06**
TP	Date	6	4.64e02	7.73e01	3.70e00	**7.82e−03**
TP	treatment:Date	12	3.57e02	2.97e01	1.42e00	2.14e−01
TKN	treatment	2	4.03e05	2.01e05	2.33e01	**1.11e−06**
TKN	Date	6	9.17e04	1.53e04	1.77e00	1.43e−01
TKN	treatment:Date	12	2.68e05	2.23e04	2.58e00	**1.89e−02**
TN	treatment	2	3.54e05	1.77e05	2.16e01	**2.10e−06**
TN	Date	6	1.05e05	1.74e04	2.13e00	8.12e−02
TN	treatment:Date	12	2.33e05	1.94e04	2.37e00	**2.92e−02**
TN:TP	treatment	2	3.16e02	1.58e02	1.73e00	1.95e−01
TN:TP	Date	6	6.80e02	1.13e02	1.24e00	3.15e−01
TN:TP	treatment:Date	12	2.86e02	2.38e01	2.61e−01	9.91e−01
Bold indicate significance. All statistics are run as two-way ANOVA.						

Supplemental Table 2. All statistical results for the mesocosm nutrient concentrations . Bold indicates significance and all measurements were run as two-way ANOVA with interactions.

Mesocosm nutrient results									
Date	Treatment	Ammonium	Nitrite	DRP	Nitrate	TP	TKN	TN	TN:TP
8/27/2021	Control	1.55±0	0.1±0	1.66±0	0.01±0	12.11±0	318.18±0	318.29±0	26.29±0
8/27/2021	Stagnant Water	9.58±8.23	0.09±0.04	4.22±0.82	0±0	15.88±3.85	409.9±45.99	409.91±45.98	26.3±3.05
8/27/2021	Water Circulation	0.45±0.21	0.07±0.03	2.46±1.61	0±0	14.25±1.67	375.48±30.17	375.48±30.17	26.44±1.21
9/3/2021	Control	0±0	0±0	1.31±0	0±0	12.05±0	307.92±0	307.92±0	25.56±0
9/3/2021	Stagnant Water	0.84±0.83	0.04±0.01	1.68±2.46	0±0	19.69±3.8	476.13±45.59	476.14±45.59	24.6±3.75
9/3/2021	Water Circulation	86.53±59.36	0.36±0.47	2.25±1.65	0.05±0.08	9.54±2.04	323.11±12.59	323.49±12.65	35.02±7.95
9/10/2021	Control	0±0	0.02±0	0.56±0	0±0	11.35±0	303.47±0	303.47±0	26.73±0
9/10/2021	Stagnant Water	4.97±7.49	0.15±0.05	3.42±3.98	0.08±0.14	19.76±7.09	523.85±81.33	524.04±81.43	27.82±5.5
9/10/2021	Water Circulation	74.08±43.41	39.71±63.97	2.36±1.53	2.64±4.28	9.99±4.68	276.17±73.96	318.52±19.98	39.27±23.74
9/17/2021	Control	0.5±0	0.02±0	0.55±0	0±0	10.61±0	255.49±0	255.49±0	24.09±0
9/17/2021	Stagnant Water	97.39±84.76	0.15±0.16	6.32±5.79	0.01±0.02	23.02±6.56	514.63±175.35	514.74±175.3	22.96±7.58
9/17/2021	Water Circulation	32.86±21.16	5.01±5.63	2.27±0.79	28.21±48.67	9.57±2.57	189.21±34.36	222.45±19.9	24.95±9.74
9/24/2021	Control	0.64±0	0.49±0	0.9±0	8.81±0	8.1±0	198.4±0	207.71±0	25.64±0
9/24/2021	Stagnant Water	339.51±192.34	0.11±0.04	14.72±9.61	0.09±0.1	27.5±6.86	650.09±72.01	650.29±71.92	24.3±3.91
9/24/2021	Water Circulation	104.77±67.93	12.78±20.92	4.48±1.09	18.3±31.64	11.98±4.64	233.82±71.57	265.04±19.16	23.99±7.26
10/1/2021	Control	0±0	0.04±0	0.53±0	0±0	9.54±0	270.72±0	270.75±0	28.38±0
10/1/2021	Stagnant Water	2.38±3.91	0.05±0.06	0.48±0.83	0.03±0.04	9.66±2.34	268.4±15.16	268.47±15.23	28.63±5.19
10/1/2021	Water Circulation	0.25±0.34	8.23±14.16	0.03±0.03	2.97±5.06	9.13±1.32	333.39±112.9	344.58±132.11	37.06±9.27
10/8/2021	Control	0.32±0	0.03±0	0.61±0	0±0	9.27±0	233.78±0	233.78±0	25.23±0
10/8/2021	Stagnant Water	3.36±5.26	2.32±3.98	1.9±2.07	0.38±0.67	12.97±3.29	386.72±38.13	389.43±42.57	30.84±4.99
10/8/2021	Water Circulation	16.93±18.39	4.72±4.92	0.28±0.17	2.31±3.14	7.78±4.33	242.37±122.98	249.42±130.25	32.62±2.39
10/22/2021	Control	1.73±0	0.04±0	0.26±0	0±0	8.71±0	318.42±0	318.43±0	36.57±0
10/22/2021	Stagnant Water	35.83±28.3	8.94±13.7	3.15±2.45	5.12±6.05	11.44±5.38	334.68±141.72	348.75±131.38	32.64±8.99
10/22/2021	Water Circulation	82.91±108.22	1.69±1.73	3.57±2.51	2.34±3.43	8.5±4.24	269.11±121.92	273.15±122.02	34.89±12.68

Supplemental Table 3. Mesocosm nutrient concentrations with their standard deviations.

Mesocosm pairwise chlorophyll-a results<br>Pairwise comparisons adjusted with Tukey's HSD					
Measurement	Comparisons	Diff	Lwr	Upr	Adjusted P
Chlorophyll-a concentration	Stagnant Water-Control	116.5175	5.406303	227.628697	**3.76e−02**
Chlorophyll-a concentration	Water Circulation-Control	-106.4717	-217.582864	4.639531	6.33e−02
Chlorophyll-a concentration	Water Circulation-Stagnant Water	-222.9892	-301.556648	-144.421685	**4.57e−09**
Bold indicate significance. All statistics are run as two-way ANOVA.					

Supplemental Table 4. Pairwise two-way ANOVA results for chlorophyll-a concentrations.

Mesocosm microcystin results						
Measurement	Comparisons	DF	SS	MS	F stat	P
Particulate	treatment	2	6.85e01	3.43e01	5.90e01	**7.79e−08**
Particulate	Date	4	4.79e01	1.20e01	2.06e01	**5.90e−06**
Particulate	treatment:Date	8	1.13e02	1.41e01	2.43e01	**3.00e−07**
Dissolved	treatment	2	3.27e−01	1.64e−01	9.80e−01	3.98e−01
Dissolved	Date	4	7.28e−01	1.82e−01	1.09e00	3.97e−01
Dissolved	treatment:Date	8	1.90e00	2.38e−01	1.42e00	2.65e−01
Total Toxins	treatment	2	3.66e01	1.83e01	2.09e00	1.36e−01
Total Toxins	Date	4	2.46e01	6.14e00	7.01e−01	5.96e−01
Total Toxins	treatment:Date	8	6.47e01	8.09e00	9.23e−01	5.07e−01

Bold indicate significance. All statistics are run as two-way ANOVA.

Supplemental Table 5. Two-way ANOVA results for microcystin concentrations in the various forms measured.

Mesocosm infection prevelance results						
Measurement	Comparisons	DF	SS	MS	F stat	P
Infection Prevelance	Treatment	2	7.89e00	3.94e00	1.64e01	**8.85e−07**
Infection Prevelance	Date	7	1.49e01	2.13e00	8.84e00	**3.35e−08**
Infection Prevelance	Treatment:Date	14	2.05e01	1.47e00	6.10e00	**2.83e−08**

Bold indicate significance. All statistics are run as two-way ANOVA.

Supplemental Table 6. Two-way ANOVA results for infection prevalence.

Mesocosm infection prevelance results					
Pairwise comparisons adjusted with Tukey's HSD					
Measurement	Comparisons	Diff	Lwr	Upr	Adjusted P
Infection Prevelance	Stagnant Water-Control	0.52514577	0.1876576	0.8626339	**1.05e−03**
Infection Prevelance	Water Circulation-Control	-0.01465694	-0.3521451	0.3228312	9.94e−01
Infection Prevelance	Water Circulation-Stagnant Water	-0.53980271	-0.7784429	-0.3011625	**1.72e−06**
Bold indicate significance. All statistics are run as two-way ANOVA.					

Supplemental Table 7. Pairwise comparisons of the two-way ANOVA results for infection prevalence.

Mesocosm infection prevelance results						
Grouping comparison indicates infections at temperatures between 19-23 or outside of that range						
Measurement	Comparisons	DF	SS	MS	F stat	P
Infection Prevelance vs temperature	Grouping	1	9.78e00	9.78e00	2.06e01	**1.52e−05**
Infection Prevelance vs temperature	Date	7	5.88e00	8.40e−01	1.77e00	1.01e−01
Bold indicate significance. All statistics are run as one-way ANOVA.						

Supplemental Table 8. Two-way ANOVA results for infection prevalence within 19-23°C and outside, labeled as Grouping.

Mesocosm qPCR results						
Measurement	Comparisons	DF	SS	MS	F stat	P
Planktothrix	treatment	2	1.12e18	5.61e17	5.00e00	**9.54e−03**
Planktothrix	date	6	4.90e18	8.17e17	7.27e00	**5.49e−06**
Planktothrix	treatment:date	11	2.05e18	1.86e17	1.66e00	1.04e−01
Rhizophydiales	treatment	2	9.58e19	4.79e19	3.70e00	**2.99e−02**
Rhizophydiales	date	6	2.34e20	3.90e19	3.01e00	**1.15e−02**
Rhizophydiales	treatment:date	11	2.94e20	2.67e19	2.07e00	**3.52e−02**
Bold indicate significance. All statistics are run as two-way ANOVA.						

Supplemental Table 9. Two-way ANOVA results for qPCR for both host (Planktothrix) and parasite (*Rhizophdiales*). Open water samples were excluded from analysis because those sample dates didn't line up with the mesocosm sample date skewing the date component of the analysis.

Mesocosm qPCR results

Pairwise comparisons adjusted with Tukey's HSD

Measurement	Comparisons	Diff	Lwr	Upr	Adjusted P
Planktothrix	Stagnant Water-Control	300322365	32497105	568147626	**2.43e−02**
Planktothrix	Water Circulation-Control	346205076	80147658	612262494	**7.46e−03**
Planktothrix	Water Circulation-Stagnant Water	45882711	-140989878	232755299	8.27e−01
Rhizophydiales	Stagnant Water-Control	2213928301	-660402328	5088258931	1.63e−01
Rhizophydiales	Water Circulation-Control	99631590	-2755726353	2954989533	9.96e−01
Rhizophydiales	Water Circulation-Stagnant Water	-2114296711	-4119834220	-108759203	**3.65e−02**

Bold indicate significance. All statistics are run as two-way ANOVA.

Supplemental Table 10. Pairwise comparisons of the two-way ANOVA results for qPCR analysis for both host (Planktothrix) and parasite (*Rhizophdiales*). Open water samples were excluded from analysis because those sample dates didn't line up with the mesocosm sample date skewing the date component of the analysis.

Mesocosm shannon diversity results

Pairwise comparisons adjusted with Tukey's HSD

Measurement	Comparisons	Diff	Lwr	Upr	Adjusted P
Shannon Diversity	Stagnant Water-Control	-1.4934340	-2.4048079	-0.5820600	**2.42e−03**
Shannon Diversity	Water Circulation-Control	-1.0909961	-2.0023701	-0.1796221	**1.96e−02**
Shannon Diversity	Water Circulation-Stagnant Water	0.4024378	-0.3218487	1.1267244	3.33e−01

Bold indicate significance. All statistics are run as two-way ANOVA.
Statistics were ran without open water samples.

Supplemental Table 11. Alpha diversity pairwise two-way ANOVA results. Open water samples were excluded from analysis because those sample dates didn't line up with the mesocosm sample date skewing the date component of the analysis.

Mesocosm community results						
Statistics ran without open water samples						
Measurement	Comparisons	DF	SS	R squared	F model	P
Beta Diversity	Treatment	2	2.80e00	2.92e−01	7.33e00	**1.00e−05**
Beta Diversity	date	6	1.92e00	2.01e−01	1.68e00	**2.97e−02**
Beta Diversity	Treatment:date	11	2.56e00	2.68e−01	1.22e00	1.71e−01
Bold indicate significance. All statistics are run using Adonis (non-parametric permutation based MANOVA).						

Supplemental Table 12. Beta diversity two-way ANOVA results. Open water samples were excluded from analysis because those sample dates didn't line up with the mesocosm sample date skewing the date component of the analysis.

Mesocosm shannon diversity 18S results					
Pairwise comparisons adjusted with Tukey's HSD					
Measurement	Comparisons	Diff	Lwr	Upr	Adjusted P
Shannon Diversity	Stagnant Water-Control	0.2510593	-0.6971902	1.199309	7.64e−01
Shannon Diversity	Water Circulation-Control	0.5174985	-0.4307510	1.465748	3.45e−01
Shannon Diversity	Water Circulation-Stagnant Water	0.2664392	-0.4871531	1.020031	6.25e−01
Bold indicate significance. All statistics are run as two-way ANOVA. Statistics were ran without open water samples.					

Supplemental Table 13. Alpha diversity of 18S pairwise two-way ANOVA results. Open water samples were excluded from analysis because those sample dates didn't line up with the mesocosm sample date skewing the date component of the analysis.
